# Integrated regulation triggered by a cryophyte ω-3 desaturase gene confers multiple-stress tolerance in tobacco

**DOI:** 10.1093/jxb/ery050

**Published:** 2018-02-08

**Authors:** Yulan Shi, Xiule Yue, Lizhe An

**Affiliations:** 1 Extreme Stress Resistance and Biotechnology Laboratory, Northwest Institute of Eco-Environment and Resources, Chinese Academy of Sciences, Lanzhou, PR China; 2 School of Life Sciences, Lanzhou University, Lanzhou, PR China

**Keywords:** ω-3 FAD gene, Ca^2+^ signaling, *Chorispora bungeana*, multiple-stress tolerance, ROS, stress-responsive gene

## Abstract

ω-3 fatty acid desaturases (FADs) are thought to contribute to plant stress tolerance mainly through linolenic acid (C18:3)-induced membrane stabilization, but a comprehensive analysis of their roles in stress adaptation is lacking. Here, we isolated a microsomal ω-3 FAD gene (*CbFAD3*) from a cryophyte (*Chorispora bungeana*) and elucidated its functions in stress tolerance. *CbFAD3*, exhibiting a high identity to Arabidopsis *AtFAD3*, was up-regulated by abiotic stresses. Its functionality was verified by heterogonous expression in yeast. Overexpression of *CbFAD3* in tobacco constitutively increased C18:3 in both leaves and roots, which maintained the membrane fluidity, and enhanced plant tolerance to cold, drought, and salt stresses. Notably, the constitutively increased C18:3 induced a sustained activation of plasma membrane Ca^2+^-ATPase, thereby, changing the stress-induced Ca^2+^ signaling. The reactive oxygen species (ROS) scavenging system, which was positively correlated with the level of C18:3, was also activated in the transgenic lines. Microarray analysis showed that *CbFAD3*-overexpressing plants increased the expression of stress-responsive genes, most of which are affected by C18:3, Ca^2+^, or ROS. Together, *CbFAD3* confers tolerance to multiple stresses in tobacco through the C18:3-induced integrated regulation of membrane, Ca^2+^, ROS, and stress-responsive genes. This is in contrast with previous observations that simply attribute stress tolerance to membrane stabilization.

## Teaser

A ω-3 fatty acid desaturase gene from *Chorispora bungeana* confers multi-stress tolerance by the integrated regulation of membrane, [Ca^2+^]_cyt_, ROS, and stress-responsive genes in tobacco

## Introduction

Environmental stresses, such as low temperature, drought, and salinity, severely limit plant growth and productivity. To withstand these abiotic stresses, plants have evolved both constitutive and inducible mechanisms that prevent or reduce adverse effects. As the outer boundary of plant cells, the cell membrane is the primary sensor of environmental stresses, and its stabilization is required for the survival of the plant (Zhang *et al.*, 2005; Shi *et al.*, 2008). Membrane stabilization, especially the maintenance of its integrity and function, is affected by lipid composition and the degree of fatty acid desaturation (Mikami and Murata, 2003; Shi *et al.*, 2008). Therefore, fatty acid desaturation caused by fatty acid desaturases (FADs), represented mainly by an increase in linolenic acid (C18:3), is considered as one of the factors involved in the tolerance of plants to many environmental stresses (Zhang *et al.*, 2005; Upchurch, 2008).

Three ω-3 FADs that catalyse the conversion of linoleic acid (C18:2) to C18:3 have been identified in Arabidopsis: two are plastidial desaturases, FAD7 and FAD8, and one is a microsomal desaturase, FAD3. It is widely accepted that ω-3 FADs are involved in plant tolerance to various abiotic stresses. In tobacco, overexpression of *AtFAD7* was reported to enhance cold tolerance (Kodama *et al.*, 1995), whereas its antisense expression reduced salt and drought tolerance (Im *et al.*, 2002). Expression of *FAD8* was induced by low temperature in Arabidopsis (Román *et al.*, 2015) and by salt treatment in maize roots (Berberich *et al.*, 1998). *FAD3* family members were up-regulated in the leaves of lima bean and soybean by drought (Zhang *et al.*, 2011) and cold (Román *et al.*, 2012), respectively. Overexpression of *LeFAD3* in tomato conferred tolerance to chilling (Yu *et al.*, 2009) and salinity stress (Wang *et al.*, 2014*a*). Although previous studies have observed the role of ω-3 FADs in stress tolerance, the relevance of these proteins in stress tolerance has always focused on the C18:3-induced membrane stabilization. However, stress tolerance is a complex process that consists of a series of responses at different levels (Perez and Brown, 2014) and cannot be achieved by membrane stabilization alone. Therefore, our knowledge of how ω-3 FADs respond to environmental stresses is still limited.

It is known that stresses trigger a rapid increase in cytosolic Ca^2+^ ([Ca^2+^]_cyt_), and the excess [Ca^2+^]_cyt_ will result in aggregation of proteins/nucleic acids and precipitation of phosphates, together with disintegration of membrane lipids, leading to cell death (Case *et al.*, 2007; Huda *et al.*, 2013*b*). Therefore, increased export of Ca^2+^ from the cell/intracellular organelles is needed to maintain [Ca^2+^]_cyt_ balance and adapt to the changing environment (Dodd *et al.*, 2010; Huda et al., 2013a, b). As one important exporter, the plasma membrane (PM) Ca^2+^-ATPase participates in various stress responses through generating a stress-induced Ca^2+^ signature (Huda *et al.*, 2013a, b; Sun *et al.*, 2016). Considering its location, we asked whether this enzyme is affected by membrane unsaturation. The same question is raised for another important membrane-bound protein, PM H^+^-ATPase, which contributes to nutrient transport by generating an electrochemical gradient (Haruta and Sussman, 2012) and indirectly drives other membrane-bound transporters (Palmgren, 2001).

Stresses also trigger a reactive oxygen species (ROS) burst, which is partially attributed to the [Ca^2+^]_cyt_ elevation (Baxter *et al.*, 2014; Perez and Brown, 2014)_._ The excess ROS accumulation will cause cell damage through lipid/protein oxidation and nucleic acid degradation (Gill and Tuteja, 2010; Huda *et al.*, 2013*a*). Therefore, an enhanced ROS-scavenging ability is required for plant stress tolerance. In fact, overexpression of a microsomal ω-3 FAD gene in tomato enhanced the activity of antioxidant enzymes and conferred tolerance to salinity stress (Wang *et al.*, 2014*a*), but the mechanism of the enhancement was not clear.

On the other hand, non-toxic levels of [Ca^2+^]_cyt_ and ROS are regarded as key players in plant stress signaling; they induce the expression of stress-responsive genes through signal transduction and amplification, and this results in stress tolerance (Dodd *et al.*, 2010; Huda *et al.*, 2013a;Baxter *et al.*, 2014; Perez and Brown, 2014). In addition, exogenous C18:3 can modulate the expression of stress-responsive genes, especially mediated by ROS (Mata-Pérez *et al.*, 2015), but there is no related study on endogenous C18:3.

*Chorispora bungeana* is a perennial crucifer inhabiting periglacial regions at altitudes of 3800–3900 m. Its growing environment is characterized by low temperatures and freeze–thaw conditions, lack of oxygen, high ultraviolet light, strong wind, and drought stress. Being closely related to Arabidopsis (Zhao *et al.*, 2012), *C. bungeana* is good plant material for the study of abiotic stress. Previous studies have confirmed that certain physiological and molecular mechanisms, rather than the existence of special morphological characteristics, might contribute towards its high survival under severe environmental conditions (Fu *et al.*, 2006; Zhang *et al.*, 2006; Shi *et al.*, 2008; Di *et al.*, 2009; Wu *et al.*, 2009; Zhang *et al.*, 2009; Zhao *et al.*, 2012). Moreover, we found that the cold tolerance of *C. bungeana* suspension-cultured cells was associated with the rapid increase in C18:3 under low temperatures (Shi *et al.*, 2008), which was produced mainly by microsomal ω-3 FAD. However, the actual mechanism for the involvement of the ω-3 FAD gene in stress tolerance is unknown because it has not yet been isolated and characterized.

In this study, we report the isolation and characterization of the microsomal ω-3 FAD gene, *CbFAD3*, from *C. bungeana*. The expression pattern and the functionality of *CbFAD3* were analysed in *C. bungeana* suspension-cultured cells and yeast cells, respectively. We also studied the function of *CbFAD3* under abiotic stresses using transgenic tobacco plants expressing *CbFAD3* under the control of the cauliflower mosaic virus (CaMV) 35S promoter. Moreover, the transcriptome of *CbFAD3*-overexpressing plants was analysed by microarray. The experimental data demonstrated that overexpression of *CbFAD3* confers tolerance to multiple stresses in tobacco plants through an integrated regulation that involves more than membrane stabilization.

## Materials and methods

### Plant material

The suspension-cultured cells and regenerated plants of *C. bungeana* were prepared as described by Shi *et al.* (2008) and Fu *et al.* (2006), respectively. About 1-cm tall seedlings were placed on half-strength Murashige and Skoog (MS) medium with 0.5 mg l^−1^ indole-3-butyric acid added for rooting. Regenerated plants having 2-cm-long roots were used for the experiments. Wild-type (WT) and transgenic tobacco (*Nicotiana benthamiana*) seeds were sterilized and germinated on MS medium. After 10-d germination, tobacco seedlings were transferred into soil (30% peat, 70% tuff), and irrigated with water every 2 d. Plants were grown at 25 °C with a 16-h photoperiod for 1–4 weeks before use.

### Experiential treatments and morphological characterization

For quantitative real-time PCR (qRT-PCR) analysis, *C. bungeana* suspension-cultured cells were exposed to 0 °C, or added to culture medium with 15% PEG6000 (−0.6 MPa) or 200 mM NaCl for various times (3, 6, 12, 24, and 48 h). For germination experiments, tobacco seeds were germinated under different temperatures (20, 18, 16, and 14 °C), or different concentrations of PEG6000 (5, 10, 15, and 17.5%) or NaCl (50, 100, 150, and 200 mM). Germination was observed at 2-d intervals up to 30 d during stress application. For survival experiments, 4-week-old tobacco plants were exposed to −2 °C for 3 d, or were not watered for 10 d, or irrigated with 300 mM NaCl for 21 d. Survival rates were measured after a 10-d period of recovery growth under normal conditions.

### Cloning and bioinformatics analysis

A 424-bp fragment of *CbFAD3* was cloned from *C. bungeana* suspension-cultured cells using degenerate primers P1 and P2 (see Supplementary Table S1 at *JXB* online), designed on the basis of a conserved domain database from tobacco, *Brassica napus*, and Arabidopsis. The 5′ and 3′ ends of *CbFAD3* were amplified using specific primers (P3–P6, Supplementary Table S1) and the SMARTer™ RACE cDNA amplification kit (Clontech, Japan). The full-length cDNA of *CbFAD3* was obtained by assembling the fragments, and the sequence was verified by PCR (using primers P7 and P8; Supplementary Table S1) and nucleotide sequencing. The sequences were analysed using Clustal X2.0 (SFI, Ireland), DNAman 5.2.2 (LynnonBiosoft, Canada), and MEGA 3.1 (ASU, USA) software or by BLAST (http://ncbi.nlm.nih.gov/blast). The nucleotide and amino acid sequences of *CbFAD3* were submitted to the NCBI GenBank database with accession numbers KM591203 and AKN35208, respectively.

### qRT-PCR analysis

The expression of *CbFAD3* in *C. bungeana* was detected using *CbACT* (AY825362) as the housekeeping gene (Di *et al.*, 2009; Wu *et al.*, 2009; Zhang *et al.*, 2009). The amplification specificity of each primer pair (P9 and P10 for *CbFAD3*, P11 and P12 for *CbACT*; Supplementary Table S2) was checked by gel electrophoresis and dissolution curve analysis. The relative gene expression (*F*) was normalized against the expression of a housekeeping gene, according to the formula:

$$\text{F}=\frac{{\left({\text{E}}_{\text{target}}\right)}^{\Delta {\text{Ct}}_{\text{target}}(control-sample)}}{{\left({\text{E}}_{\text{housekeeping}}\right)}^{\Delta {\text{Ct}}_{\text{housekeeping}}(control-sample)}}$$

which is considered as an accurate and reproducible mathematical model (Pfaffl, 2001). The amplification efficiencies (*E*) for both target and housekeeping genes were between 90 and 110%. The cycle threshold (*C*_t_) for these genes was obtained from three independent biological experiments.

### Heterogonous expression in yeast

The coding region of *CbFAD3* was cloned into pYES2.0 (Invitrogen, USA) using specific primers (P13 and P14; Supplementary Table S1), to construct the expression plasmid pYES2-*CbFAD3*. pYES2-*CbFAD3* and pYES2.0 were transformed into *Saccharomyces cerevisiae* strain INVSc1 (Invitrogen, USA) using *S. cerevisiae* EasyComp transformation kit (Invitrogen, USA). The yeast transformants were selected and cultured according to the method of Román *et al.* (2012). When the OD_600_ of the culture reached 0.2–0.3, gene expression was induced by adding 2% (w/v) galactose. Yeast cells were harvested by centrifugation at 1500 *g* for 5 min at 4 °C and washed with distilled water. The extraction and SDS-PAGE of total yeast proteins were performed as described by Horvath and Riezman (1994). The production of C18:3 was induced by adding 2% (w/v) galactose, 50 μM C18:2 (Sigma-Aldrich, USA) and 0.1% (w/v) NP-40, and was measured after growth at 20 °C for 3 d.

### Transformation and generation of transgenic plants

The coding region of *CbFAD3*, amplified using specific primers (P15 and P16; Supplementary Table S1), was cloned within the *Xba*I–*Sac*I site of the binary vector pBI121 to replace the *GUS* gene and construct the recombinant plasmid, pBI121-*CbFAD3*, under the control of the CaMV 35S promoter. The pBI121-*CbFAD3* plasmid was introduced into *Agrobacterium tumefaciens* strain GV3101 by electroporation. The transgenic tobacco plants were generated using the *Agrobacterium*-mediated transformation method (Horsch, 1985). Five positive transgenic lines exhibiting 3:1 segregation ratio were identified by PCR using primers P17 and P18 (Supplementary Table S1; Supplementary Fig. S1A). The homozygous lines were obtained by backcrossing or self-pollination, and the expression level of *CbFAD3* was verified by qRT-PCR using primers P9 and P10 (Supplementary Table S2; Supplementary Fig. S1B). *NtL25* (L18908) was used as the housekeeping gene (Schmidt and Delaney, 2010) using primers P19 and P20 (see Supplementary Table S2). Three independent homozygous T_3_ transgenic lines (L2, L3, and L4) showing higher expression levels were used in the experiments.

### Extraction and analysis of fatty acids

Lipids and total fatty acids were extracted from 5 g of tobacco leaves or roots as described in our previous report (Shi *et al.*, 2008). The total lipid and fatty acid composition of whole yeast cells was determined using the one-step method of Garcés and Mancha (1993). Fatty acid methyl esters of each sample were analysed with a gas chromatograph–mass spectrometer (6890N-5975C; Agilent, USA) fitted with a capillary column (Agilent DB-FFAP; 30 m×0.25 mm×0.5 µm). Hydrogen was used as a carrier gas with a linear rate of 1.1 ml min^−1^ and split ratio of 100:1. The injector and detector temperature was 200 °C, and the column temperature was programmed to increase from 70 to 230 °C (10 min holding) at a rate of 8 °C min^−1^. The voltage of the ionization source was 70 eV along with a solvent delay of 1.5 min. The ion source, quadrupole, and interface temperature were 230, 150, and 250 °C, respectively.

### Measurement of electrolyte leakage, chlorophyll fluorescence, malondialdehyde, H_2_O_2_, and antioxidant enzymes

Electrolyte leakage from tobacco leaves was measured using a conductivity meter (Mettler-Toledo, Swizerland) as described in our previous publication (Shi *et al.*, 2008). The maximum efficiency of photosystem II photochemistry (*F*_v_/*F*_m_) of fully expanded leaves was measured using a PAM-2100 fluorometer (Walz, Germany). The contents of malondialdehyde (MDA) and H_2_O_2_ of tobacco leaves were determined as described previously (Yang *et al.*, 2012). To determine the activities of superoxide dismutase (SOD), catalase (CAT), and peroxidase (POD), tobacco leaves (0.5 g) were ground in 50 mM KH_2_PO_4_ buffer (pH 7.8, containing 1% polyvinylpyrrolidone) at 0 °C. The homogenate was centrifuged at 15 000 *g* for 20 min at 4 °C. The supernatant was used to determine the enzyme activity according to the method described by Miao *et al.* (2010).

### Preparation of plasma membrane and analysis of membrane fluidity and enzymes

PMs were isolated from 10 g of tobacco leaves or roots by aqueous two-phase partitioning as described in our previous report (Shi *et al.*, 2008). The purity of PM vesicles was estimated according to the method of Widell and Larsson (1990). The enzymatic activity inhibited by vanadate, azide, nitrate, and molybdate was about 85, 1.6, 4.9, and 2.3%, respectively, indicating that the PM vesicles were well purified. Protein content was determined by a dye–protein binding method using bovine serum albumin as a standard (Bradford, 1976). According to the protocols described in our previous publication (Shi *et al.*, 2008), the fluidity of isolated PM was measured using a fluorescent probe (1,6-diphenyl-1,3,5-hexatriene, Sigma-Aldrich). The activity of PM H^+^-ATPase was determined by monitoring P_i_ release at 660 nm. The activity of PM Ca^2+^-ATPase was measured as eosin-sensitive Mg-inosine triphosphate hydrolysis (Giacometti *et al.*, 2012).

### Measurements of Ca^2+^ fluxes

The net flux of Ca^2+^ was measured using a non-invasive micro-test technology (NMT, Younger USA Science & Technology Corp., USA). One-week-old seedlings of WT and transgenic tobacco grown in backfilling solution (0.05 mM CaCl_2_, 0.1 mM KCl, 0.1 mM MES, pH 6.0) were fixed under a microscope. A Ca^2+^-selective microelectrode (3 mm aperture, XYPG120-2) was propelled to approximately 300–400 μm from the root apex. Steady-state Ca^2+^ fluxes were measured for 4 min. Then, PEG6000 and NaCl were applied to a final concentration of 15% and 200 mM, respectively, and measurements were taken for another 10 min. The data measured during the first minute were discarded because of the diffusion effects of stock addition. The concentration gradients were measured by moving the microelectrode a distance of 30 μm in 5 s. The corrected slope and intercept of micropipette was 26.32 and 33.33, respectively.

### Protoplast isolation and [Ca^2+^]_cyt_ measurement

Protoplasts were isolated from tobacco root epidermis using the enzymatic digestion procedure described by Rutschow *et al.* (2014). The protoplast suspensions were then incubated with Fluo-3 AM (Sigma-Aldrich) at a final concentration of 5 μM at 30 °C for 45 min and washed with MMG solution (0.4 M mannitol, 15 mM MgCl_2_, 4 mM MES, pH 5.7) containing 2 mM CaCl_2_. The pre-incubated protoplast suspension was put in the middle of a poly-L-lysine-pretreated (0.003% w/v) slide to fix individual protoplasts. The average fluorescence of the Fluo-3-Ca^2+^ conjugate from a single protoplast was measured at 511 nm using laser scanning confocal microscopy (LSCM; Leica SP8, Germany) under an excitation wavelength of 488 nm. When PEG solution and NaCl solution were added to a final concentration of 15% and 200 mM, respectively, the detection started and continued at 3-s intervals for 5 min.

### Microarray analysis

Two-week-old tobacco seedlings were treated with 200 mM NaCl for 6 h. Leaves from five independent plants were pooled as one biological sample, and three samples were hybridized separately for each line. The samples were sent to CapitalBio Corporation (Beijing, China) for microarray analysis. A microarray chip for *N. tabacum* (4 × 44 format; G2514F; Agilent) was scanned by an Agilent G2565CA microarray scanner, and analysed by GeneSpring GX software. The *P*-value was calculated with Student’s unpaired *t*-test and corrected for false discovery rate (Benjamini–Hochberg). A total of 43 817 genes from the tobacco genome were detected; 351 genes exhibiting more than 2-fold enhancement (115 genes) or reduction (236 genes) in the transcript levels were considered to show significant alterations in expression. The raw data files were deposited in the NCBI GEO database with accession number GSE74260 (GSM1915764-1915769). To validate the microarray data, the increased expression of six genes was compared between transgenic and WT tobacco plants by qRT-PCR (see Supplementary Fig. S2) using specific primers (P21–P32; Supplementary Table S2). *NtL25* (L18908) was used as the housekeeping gene (Schmidt and Delaney, 2010) using primers P19 and P20 (see Supplementary Table S2).

### Statistics

All data were analysed for variance using SPSS 13.0 (SPSS Inc., Chicago, IL, USA), and statistical significance between samples was indicated when *P*<0.05.

## Results

### Isolation and analysis of *CbFAD3* from *C. bungeana*

**Fig. 1.**
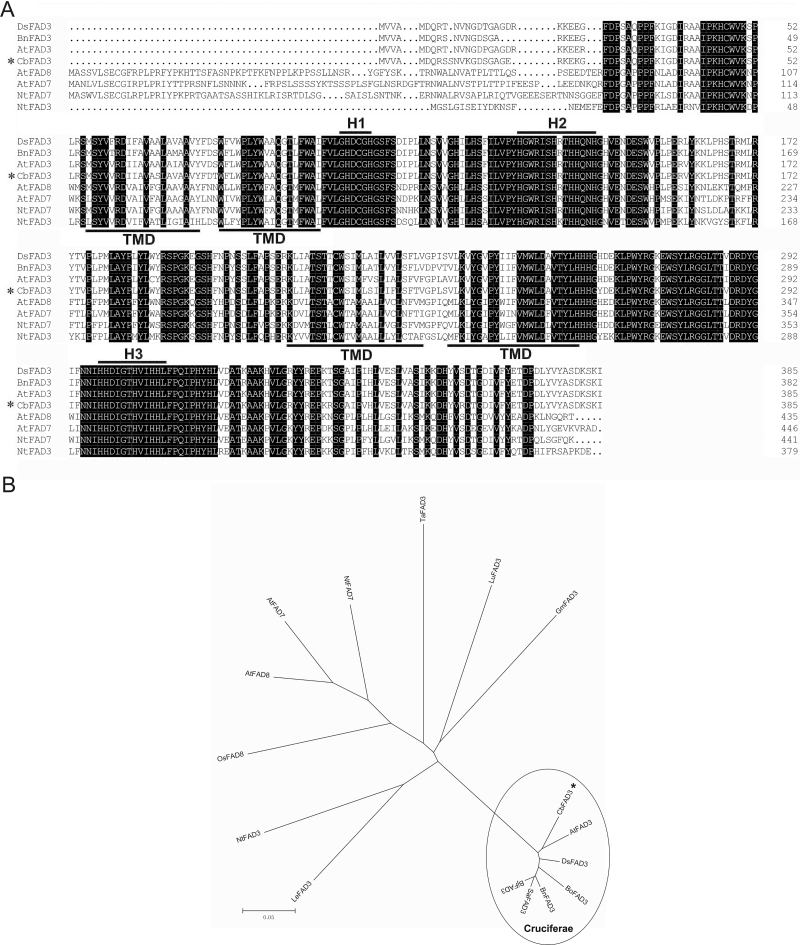
Sequence analysis of CbFAD3 and ω-3 FADs in various plant species. (A). Amino acid alignment of CbFAD3 and other ω-3 FADs from selected plant species. The sequence were aligned and displayed using Clustal X and DNAman software, respectively. Identical residues is shown on a background of black. The three conserved histidine clusters (H1–H3) are overlined, and the four transmembrane domains (TMD) are underlined. (B). Phylogenetic tree analysis of CbFAD3 and other FAD family members from selected plant species. Alignments were calculated with Clustal X software, and the analysis was performed using the neighbor-joining method implemented in the MEGA package. Poisson correction was used for multiple substitutions, and the bootstrap value was 1000 replicates. The position of CbFAD3 is indicated by an asterisk.

After full length verification, the complete *CbFAD3* cDNA was obtained, having 1493 bp and an 1161-bp open reading frame (ORF) from 152 bp to 1312 bp. The ORF encodes a deduced protein of 387 aa, with a predicted molecular mass of 44.2 kDa and a p*I* value of 8.92. CbFAD3 contained four conserved transmembrane domains (TMD; Fig. 1A) and three histidine boxes (H1–H3; Fig. 1A), which are considered as highly conserved in all the membrane-bound FADs and ω-3 FADs, respectively (Los and Murata, 1998). The sequence analysis data showed that CbFAD3 exhibited high identity to other cruciferous microsomal ω-3 FADs, such as DsFAD3 (94%), AtFAD3 (93%), BnFAD3 (92%), BjFAD3 (92%), BoFAD3 (92%), and SaFAD3 (92%). The phylogenetic tree showed that CbFAD3 was clustered in the same clade as the cruciferous FAD3s, and was closest to Arabidopsis AtFAD3 (Fig. 1B). These analyses indicate that *CbFAD3* is a microsomal ω-3 FAD gene.

### Expression pattern of *CbFAD3* in *C. bungeana*

**Fig. 2.**
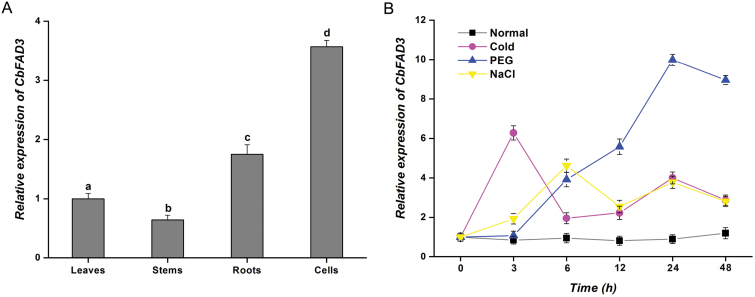
qPCR analysis for the expression of *CbFAD3* in *C. bungeana.* (A) Expression of *CbFAD3* in different tissues. The cDNAs for analysis were prepared from the regenerated plants and the cultured cells of *C. bungeana.* Data were calibrated relative to the gene expression level in leaves, which was set at a value of 1. (B) Expression of *CbFAD3* is induced by cold, drought, and salt stresses. The suspension-cultured *C. bungeana* cells were exposed to 0 °C, or treated with 15% PEG or 200 mM NaCl. The cDNAs were prepared from the cultured cells at various treatment times (3, 6, 12, 24, and 48 h). Data were calibrated relative to the gene expression level before treatment (0 h), which was set at a value of 1. *CbACT* was used as the internal control. Values are means ±SE of three biological replicates for each experiment. Statistical significance between samples, determined by Student’s *t*-test, is indicated by different letters.

To analyse the expression of *CbFAD3* in *C. bungeana*, qRT-PCR was performed. The data showed that *CbFAD3* was expressed in all the tested tissues, with the highest expression in suspension-cultured cells, followed by that in roots and leaves, with the lowest expression observed in stems (Fig. 2A). This expression pattern is similar to that of *AtFAD3* in Arabidopsis (Kodama *et al.* 1997). Using *C. bungeana* suspension-cultured cells, we found that the expression of *CbFAD3* was significantly induced by various abiotic stresses, whereas there was no significant variation in the gene expression under normal conditions (Fig. 2B). The induced expression of *CbFAD3* peaked at 3, 6, and 24 h under cold (0 °C), NaCl (200 mM), and PEG (15%) treatments, respectively, with the corresponding maximum increases of 6.3-, 4.6-, and 10-fold, respectively, compared with untreated controls. Consistent with previous findings that FAD3 genes involved in stress adaptation can be induced by abiotic stresses (Yu *et al.* 2009; Zhang *et al.* 2011; Román *et al.* 2012), these results suggest that *CbFAD3* might be involved in response to cold, drought, and salt stresses.

### Expression of *CbFAD3* in yeast leads to conversion of C18:2 to C18:3

**Fig. 3.**
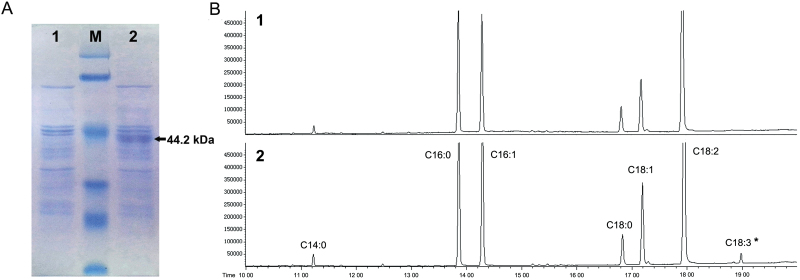
Heterogonous expression of *CbFAD3* in *S. cerevisiae*. (A) SDS-PAGE of total proteins in transgenic yeast cells grown at 20 °C. The data were measured after the induction of 2% galactose for 3 d. (B) Gas chromatogram of total fatty acids in transgenic yeast cells grown at 20 °C. The data were measured after the induction of 2% galactose, 50 µM C18:2, and 0.1% NP-40 for 3 d. Yeast cells transformed with pYES2.0 and pYES2.0-*CbFAD3* are represented as (1) and (2), respectively.

Yeast has been proven to be a suitable heterologous expression system for studying the functionality of microsomal ω-3 FAD genes (Dyer *et al.*, 2001). To determine the functionality of *CbFAD3*, the ORF of *CbFAD3* was expressed in *S. cerevisiae* under the galactose-inducible promoter of the pYES2.0 vector. The results of SDS-PAGE showed that a 44.2 kDa protein was induced in yeast cells transformed with pYES2-*CbFAD3*, but was not induced in the controls transformed with empty pYES2 vector (Fig. 3A). The fatty acid analysis of whole yeast cells clearly indicated that CbFAD3 could effect the conversion of C18:2 to C18:3 in *CbFAD3*-transformed yeast cells, whereas no C18:3 was detected in the controls (Fig. 3B). The percentage of C18:3 obtained from *CbFAD3*-transformed yeasts at 20 °C was 0.7% (see Supplementary Table S3), which was more than that (about 0.3%) from yeast-expressed Arabidopsis AtFAD3 (Kumar *et al.*, 2012). These results confirm that *CbFAD3* is a functional microsomal ω-3 FAD gene.

### Overexpression of *CbFAD3* in tobacco enhanced plant tolerance to multiple abiotic stresses

**Fig. 4.**
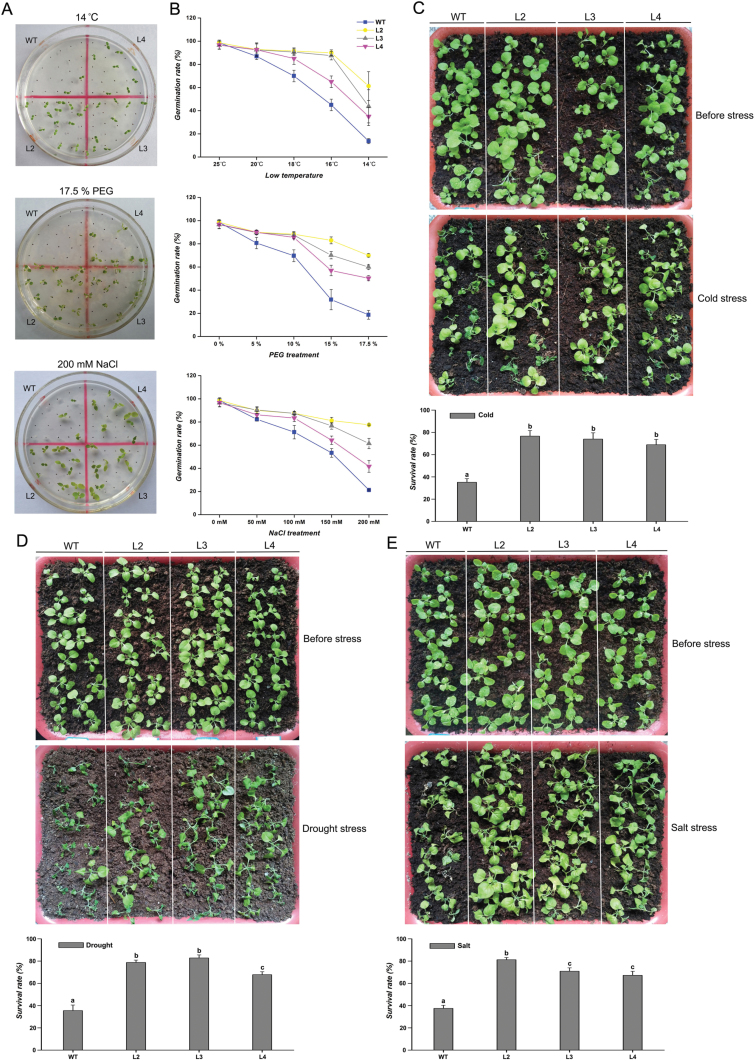
*CbFAD3*-overexpressing tobacco plants showed enhanced tolerance to multiple abiotic stresses. (A, B) Germination of transgenic and WT tobacco seeds under different treatments. Photographs were taken 24 d after low-temperature exposure and 14 d after seeding on PEG or NaCl plates. Germination rates were observed at 2-d intervals up to 30 d after treatment (*n*=100 seeds for each line from five individual plates). (C–E) Survival of transgenic and WT tobacco plants under cold, drought, and salt stresses. Plants were transferred into soil after plate germination (MS medium), and irrigated with water every 2 d. Four-week-old tobacco plants were exposed to −2 °C for 3 d, not watered for 10 d, or irrigated with 300 mM NaCl solution at 3-d intervals up to 21 d and photographed. Survival rates were measured after 10-d recovery growth under normal conditions (*n*=90 plants for each line from five individual flower pots). Transgenic lines L2, L3, and L4 were used in these experiments. Values are means ±SE from five independent trials. Statistical significance between samples, determined by Student’s *t*-test, is indicated by different letters.

To clarify the role of *CbFAD3* in stress tolerance, the germination and survival of transgenic tobacco were observed under different abiotic stresses. Under normal conditions, there were no significant differences in germination between transgenic and WT seeds. Although seed germination was inhibited by low-temperature, PEG and NaCl treatments, the germination rates of the transgenic lines were much higher than that of WT tobacco plants (Fig. 4A, B). In the chilling temperature condition (14 °C), the germination rates were 35.0–61.3% for transgenic seeds and only about 13.8% for WT controls. Similarly, with the high-PEG (17.5%) and high-salt (200 mM) media, the germination rates of transgenic seeds were more than 50% and 40%, respectively, whereas those of WT controls were around 20%.

There were no significant differences in growth between WT and transgenic plants under normal conditions, whereas the survival rates were significantly higher in transgenic plants than in WT controls under abiotic stresses (Fig. 4C–E). Cold exposure (−2 °C for 3 d) led to WT tobacco becoming wilted and nipped, whereas it had a weaker influence on transgenic plants. After recovery, the survival rate of transgenic and WT plants was 68.9–76.7% and 35.2%, respectively. Likewise, an about 2.0-fold survival rate was also found in transgenic lines as compared with WT controls under drought (not watered for 10 d) and salt treatments (300 mM NaCl for 21 d). These findings indicate that overexpression of *CbFAD3* in tobacco can enhance plant tolerance to multiple abiotic stresses.

### *CbFAD3*-overexpressing plants had improved physiological traits under abiotic stresses

**Fig. 5.**
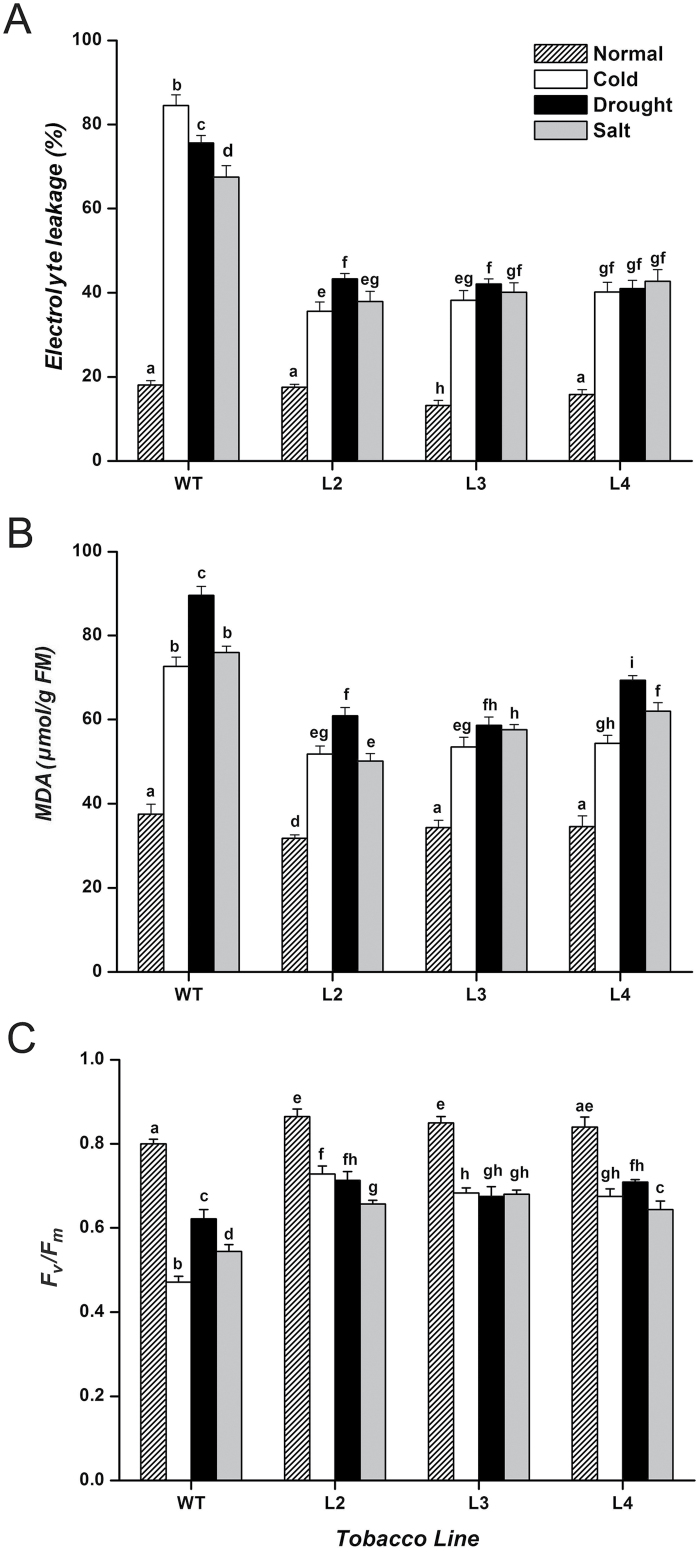
Physiological comparisons between *CbFAD3*-transgenic and WT tobacco leaves under stress conditions. (A) Electrolyte leakage, reflecting the integrity of the cellular membranes. (B) MDA content, showing the state of lipid peroxidation. (C) *F*_v_/*F*_m_ ratio, representing the photosynthetic efficiency. Transgenic lines L2, L3, and L4 were used in these experiments, and for each line more than five plants were used in every experiment. Four-week-old tobacco plants were exposed to 0 °C, not watered, or treated with 200 mM NaCl for 3 d, and then measured. Values are means ±SE of three biological replicates for each experiment. Statistical significance between samples, determined by Student’s *t*-test, is indicated by different letters.

To further confirm the enhanced stress tolerance caused by *CbFAD3*, various physiological traits important for stress responses were measured in the leaves of transgenic tobacco plants. As shown in Fig. 5, there were signs of increase in membrane stability and photosynthetic capacity in several, but not all, *CbFAD3*-overexpressing lines under normal conditions; however, these physiological traits were much better in all transgenic lines than in WT controls under abiotic stresses. During cold exposure, lower ion leakage (42.1–47.6%) and MDA accumulation (71.3–74.8%), and higher chlorophyll fluorescence (*F*_v_/*F*_m_, 143.3–154.6%) were observed in transgenic lines when compared with WT controls. Similar physiological phenomena were also observed in the transgenic plants under drought and salt treatments. These data show that *CbFAD3*-overexpressing plants demonstrate reduced damage and higher integrity of cellular membranes under various abiotic stresses.

### *CbFAD3*-overexpressing plants had constitutive high membrane unsaturation

**Fig. 6.**
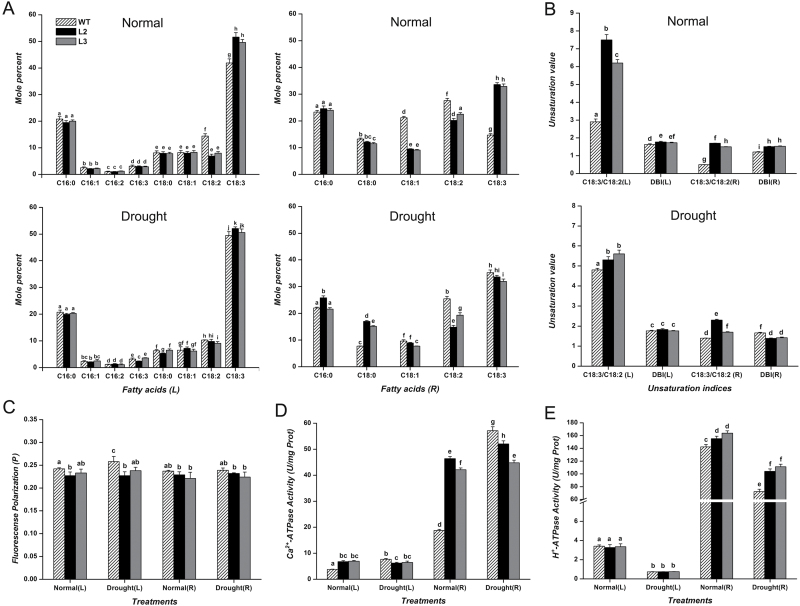
Changes in fatty acid composition, enzyme activity and membrane fluidity between *CbFAD3*-transgenic and WT tobacco plants after a 10-d drought treatment. (A) Fatty acid composition of leaves and roots. Data are expressed as molar percentages obtained from the quantitative analysis of peak area chromatogram. (B) Unsaturation indices calculated from fatty acid composition. Double bond index, DBI=[(%18:1)+2(%18:2)+3(%18:3)]/100. (C) Fluidity of plasma membrane from leaves and roots. Data are represented as fluorescence polarization (*P*). (D) Activity of PM Ca^2+^-ATPase from leaves and roots. (E) Activity of PM H^+^-ATPase from leaves and roots. Transgenic lines L2 and L3 were used in these experiments, and each line was used with more than twenty 4-week-old plants in every experiment. Data from leaves and roots are represented as L and R, respectively. Values are means ±SE of three biological replicates for each experiment. Statistical significance between samples, determined by Student’s *t*-test, is indicated by different letters.

To identify the contribution of *CbFAD3* to membrane unsaturation, the fatty acid compositions of transgenic tobacco plants were measured. Overexpression of *CbFAD3* constitutively increased the fatty acid unsaturation, including the C18:3 content, C18:3/C18:2 ratio, and double bond index (DBI), in both leaves and roots of tobacco plants (Fig. 6A, B). Under normal conditions, the average level of C18:3 in transgenic lines increased by about 20.8% in leaves and by 126.2% in roots compared with the levels in WT plants. The increases in C18:3/C18:2 ratio and DBI in transgenic lines were also greater in roots than in leaves. After 10 d of drought treatment, the levels of C18:3 in both leaves and roots of transgenic plants were almost unchanged, whereas those of WT plants increased to the levels found in transgenic lines (Fig. 6A, B). The consumption of C18:2 in WT roots was compensated by the conversion of stearic acid (C18:0) and oleic acid (C18:1) to C18:2. Although the C18:3/C18:2 ratio in transgenic lines was higher than that in WT plants, the DBI in WT plants was equal to (in leaves) or more than (in roots) that in the transgenic lines. These results indicate that overexpression of *CbFAD3* in tobacco can constitutively increase the fatty acid unsaturation, especially the level of C18:3, to the same level as that induced by drought in WT plants.

### *CbFAD3*-overexpressing plants continuously stabilized membrane fluidity and activated PM Ca^2+^-ATPase

To evaluate the function of *CbFAD3* in membrane stabilization, the membrane fluidity and enzyme activities of transgenic tobacco plants were examined. The value *P* calculated from fluorescence polarization indices is an indication of membrane fluidity (Mykytczuk *et al.*, 2007); the higher the value, the lower is the fluidity. In *CbFAD3*-overexpressing tobacco plants, the constitutive fatty acid desaturation did not significantly change the membrane fluidity, which was already optimal for survival, under normal conditions, but maintained the fluidity under drought stress (Fig. 6A–C). Moreover, the drought-induced fatty acid desaturation in WT tobacco plants also maintained the membrane fluidity under drought stress except for a slight rigidification in the leaves.

The experimental data together with a correlation analysis showed that the sustained activation of PM Ca^2+^-ATPase in leaves and roots of *CbFAD3*-overexpressing plants was exactly correlated with the constitutive accumulation of C18:3, and the inducible activation of PM Ca^2+^-ATPase in WT plants was in accordance with the drought-induced increase in C18:3 (Fig. 6A, B, E; Table 1). However, the enzyme activities in both WT and transgenic tobacco plants did not correlate with membrane fluidity. The drought-induced inhibition of PM H^+^-ATPase, which did not correlate with the membrane unsaturation and fluidity, was not different between WT and transgenic leaves, but was observably milder in the roots of transgenic lines (Fig. 6A, B, E; Table 1). These results suggest that the *CbFAD3*-induced constitutive accumulation of C18:3 might maintain membrane fluidity and activate PM Ca^2+^-ATPase under any condition, although its role in the PM H^+^-ATPase was complex.

**Table 1.** Pearson correlation coefficients (two-tailed) between membrane unsaturation indices and physiological indices in leaves (L) or roots (R) before and after drought stress

	C18:3	C18:3/C18:2	DBI
EL (L)	0.26	−0.19	0.33
MDA (L)	0.25	−0.23	0.34
*F*_v_/*F*_m_ (L)	−0.11	0.37	−0.14
MF (L)	−0.18	−0.33	0.04
MF (R)	−0.11	−0.18	0.14
Ca^2+^-ATPase (L)	0.81***	0.69**	0.63*
Ca^2+^-ATPase (R)	0.95***	0.79***	0.82***
H^+^-ATPase (L)	−0.42	0.10	−0.43
H^+^-ATPase (R)	−0.31	−0.29	−0.30
SOD (L)	0.73***	0.37	0.69**
CAT (L)	0.53*	−0.01	0.59**
POD (L)	0.45	−0.05	0.50*
H_2_O_2_ (L)	0.28	−0.19	0.36

The data from *CbFAD3*-transgenic (L2 and L3) and WT tobacco plants were used for the calculation (three replications, *n*=12 × 3). Significant positive correlation (*R*≥0.50, *P*<0.05) is indicated in bold. *P<0.05, **P<0.01, ***P<0.001.

### *CbFAD3*-overexpressing plants changed the stress-induced Ca^2+^ signaling during early stresses

To explore the role of *CbFAD3* in regulating Ca^2+^ signaling, the net Ca^2+^ flux and [Ca^2+^]_cyt_ of transgenic tobacco plants were determined under different stresses. Although both of the two Ca^2+^ indices reflect a combined result of Ca^2+^ influx through Ca^2+^ channels and Ca^2+^ efflux driven by Ca^2+^-ATPase and Ca^2+^ exchangers (Bose *et al.*, 2011), they are not completely consistent with each other. This is because the net Ca^2+^ flux detected by NMT presents a dynamic Ca^2+^ state just around the plasma membrane of cells that adjoin the microelectrode, whereas the [Ca^2+^]_cyt_ monitored using LSCM shows the cytoplasmic Ca^2+^ variation affected by the Ca^2+^ flux from both plasma membrane and tonoplast.

**Fig. 7.**
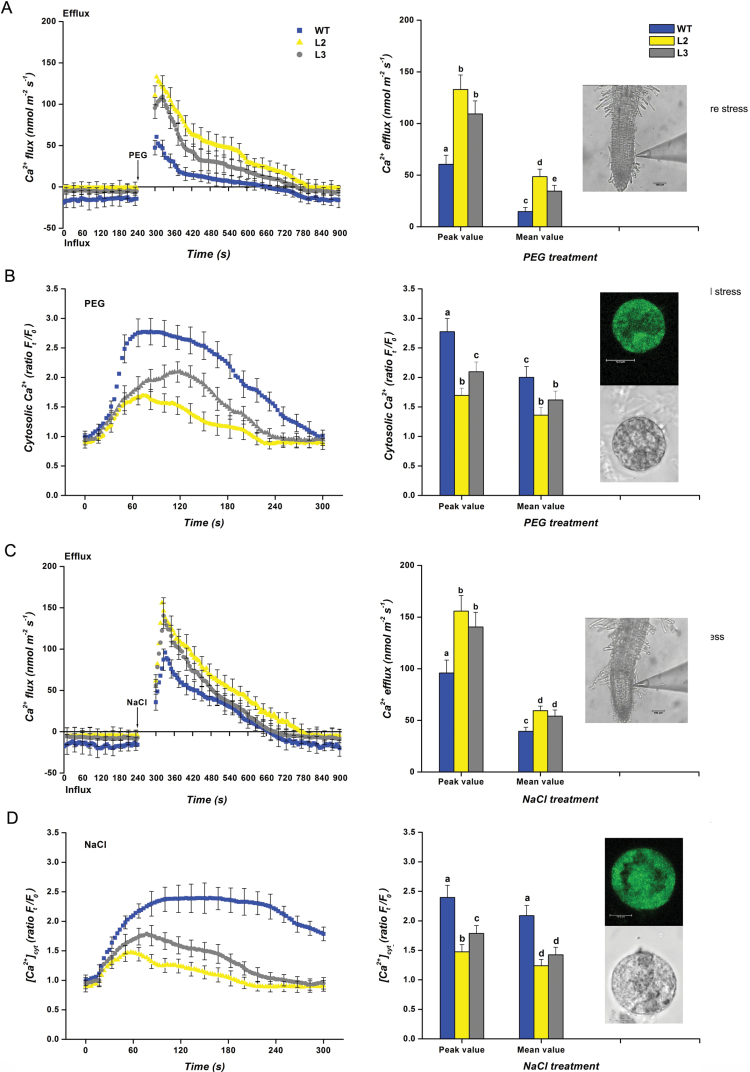
Dynamics of Ca^2+^ fluxes and [Ca^2+^]_cyt_ in *CbFAD3*-transgenic and WT tobacco plants under different treatments. (A, C) Ca^2+^ fluxes in root tips treated with 15% PEG or 200 mM NaCl (*n*=10 ten-day-old seedling roots). Values are means ±SE from 10 independent trials. The inset photomicrograph showed that the detection site was in the elongation zone epidermis of root tips. Scale bar: 100 µm. (B, D) [Ca^2+^]_cyt_ in root epidermis protoplasts treated with 15% PEG or 200 mM NaCl (*n*=30 root protoplasts). The root protoplasts were prepared from more than 100 ten-day-old seedlings for each line. The value is presented as the relative fluorescence ratio of *F*_t_/*F*_0_. Values are means ±SE of 30 independent biological trials. The inset photomicrograph showed that the Ca^2+^ level was detected from the cytoplasm of intact root protoplast. Scale bar: 100 µm. The transgenic lines L2 and L3 were used in these experiments. Statistical significance between samples, determined by Student’s *t*-test, is indicated by different letters.

When exposed to 15% PEG, tobacco root tips exhibited a transient increase in Ca^2+^ efflux, which was 0.9–1.4 times larger in transgenic lines than in WT plants (Fig. 7A). The PEG stress also induced an instantaneous [Ca^2+^]_cyt_ elevation in the root protoplasts, and the elevation in transgenic lines was only 64.0–77.8% of that in WT controls (Fig. 7B). The higher Ca^2+^ efflux (142.0–156.8%) and the lower [Ca^2+^]_cyt_ elevation (60.9–71.9%) were also observed in transgenic lines under salt shock (200 mM NaCl) (Fig. 7C, D). These results revealed that the sustained activation of the PM Ca^2+^-ATPase in *CbFAD3*-overexpressing plants changes the stress-induced Ca^2+^ signaling during early stresses.

### *CbFAD3*-overexpressing plants enhanced ROS scavenging under abiotic stresses

**Fig. 8.**
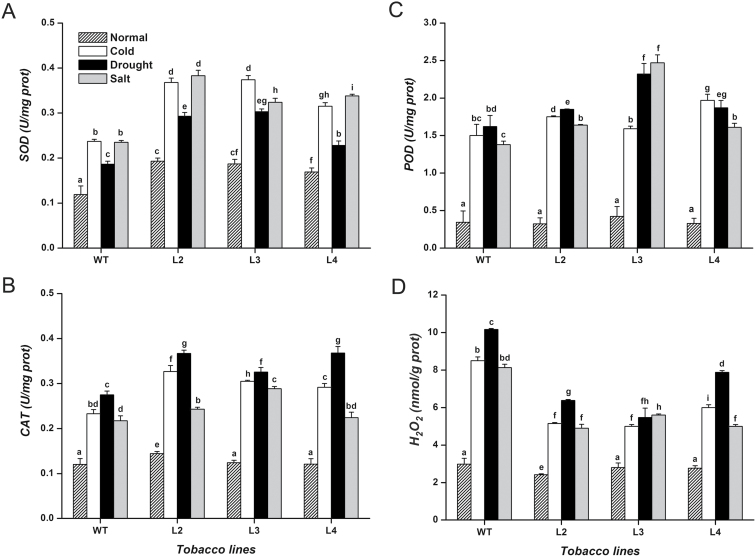
Antioxidant response to abiotic stresses in *CbFAD3*-transgenic and WT tobacco leaves. (A–C) Activities of superoxide dismutase (SOD), catalase (CAT), and peroxidase (POD), respectively. (D) Hydrogen peroxide (H_2_O_2_) content. Transgenic lines L2, L3, and L4 were used in these experiments, and for each line more than five plants were used in every experiment. Four-week-old tobacco plants were exposed to 0 °C, not watered, or treated with 200 mM NaCl for 24 h, and then measured. Values are means ±SE of three biological replicates for each experiment. Statistical significance between samples, determined by Student’s *t*-test, is indicated by different letters.

To reveal the function of *CbFAD3* in ROS scavenging, the activities of antioxidant enzymes and the level of H_2_O_2_ were analysed in the leaves of transgenic tobacco plants. Under normal conditions, the ROS scavenging ability of transgenic plants was not significantly higher than that of WT plants, except for the SOD activity. However, the activities of SOD, CAT, and POD increased rapidly in stress-treated transgenic lines compared with that in the WT controls (Fig. 8A–C). Under stress conditions, the transgenic lines showed significantly less accumulation of H_2_O_2_ and MDA, and less ion leakage, as well as obviously higher photosynthetic activities (Fig. 5A–C; Fig. 8D), confirming reduced oxidative damage during the onset of the stresses. Furthermore, the correlation coefficients verified that the activities of these antioxidant enzymes had a certain degree of positive correlation with membrane unsaturation, including the C18:3 content and DBI (Table 1).

### *CbFAD3*-overexpressing plants increased the expression of stress-responsive genes under stress condition

To elucidate the molecular mechanism of stress tolerance mediated by *CbFAD3*, increased expression of genes in transgenic plants compared with WT controls was identified under salt stress using microarray analysis. According to the search results of the NCBI database, 61 genes annotated with related functions were confirmed from the 115 genes in transgenic plants (Table 2); the others, annotated as ‘uncharacterized gene/protein’ in the database, are not shown. Among the confirmed genes, there were 59 that were directly or indirectly involved in plant stress response; these included genes for nitrogen/sulfur metabolism-related proteins (15 genes), cell wall/membrane-related proteins (11 genes), photosynthesis-related proteins (six genes), protein kinases (six genes), ROS-responsive proteins (five genes), genes for transcription factors (four genes), chromatin remodeling or DNA methylation proteins (three genes), signaling proteins (three genes), alkaloid biosynthesis-related proteins (three genes), cell cycle-related proteins (two genes), as well as one gene for disease resistance protein. Beside these, two pentatricopeptide repeat (PPR)-containing protein genes linked with the recovery of fertility were detected, which might ensure the fertility of transgenic plants. These results indicate that overexpression of *CbFAD3* in tobacco can increase the expression of stress-responsive genes under stress conditions.

**Table 2.** Transcripts significantly increased in *CbFAD3*-overexpressing tobacco seedlings compared with those of WT controls under salt stress conditions

Number	Probe name	Accession no.	Gene annotation	Fold (log_2_)	Function	Reference
1	A_95_P204097	EH618725	Cytochrome *c* oxidase	15.02	Chilling and salt tolerance	De Santis *et al.* (1999) Yan *et al.* (2005)
2	A_95_P267086	FG169445	Monocopper oxidase-like protein	14.00	Plant growth (i.e. cell wall expansion)	Sedbrook *et al.* (2002)
3	A_95_P117392	DV160317	Disease resistance protein RGA2	12.64	Defense against pathogen attack	Loutre *et al.* (2009)
4	A_95_P238494	FG167609	Asparagine synthetase	12.55	Response to abiotic stresses	Wang *et al.* (2005)
5	A_95_P113687	CV021257	Caffeoyl-CoA *O*-methyltransferase	11.31	Response to salt and water deficit stresses	Senthil-Kumar *et al.* (2010)
6	A_95_P268896	DW003469	YLS2-like protein	11.26	Response to salt stress	Aghaei and Komatsu (2013)
7	A_95_P254509	EB438355	Histidine kinase	10.89	Plant growth and stress responses	Cao *et al.* (2015)
8	A_95_P016001	EB678560	Non-specific lipid-transfer protein	10.75	Response to abiotic stresses	Gangadhar *et al.* (2016)
9	A_95_P281518	FG645498	Chromatin-remodeling complex-like	10.30	Response to drought, cold and salt stresses	Kim *et al.* (2010)
10	A_95_P035683	BP128538	Zinc finger protein	9.88	Plant growth and stress responses	Yang *et al.* (2014)
11	A_95_P234479	FG170626	Methyltransferase-like protein	9.03	Response to abiotic/biotic stresses	Sahu *et al.* (2013)
12	A_95_P054511	BP133457	Cytochrome P450 -like	5.68	Plant development and abiotic stress tolerance	Perez and Brown (2014)
13	A_95_P084005	BP528907	MYB transcription factor	5.48	Plant growth, development and stress response	Zhang *et al.* (2012)
14	A_95_P134852	EB437708	SNF1-related protein kinase	4.31	Response to salt and drought stresses	Xu *et al.* (2009)
15	A_95_P006516	EH616694	Glycine-rich cell wall structural protein	3.70	Salt tolerance and response to pathogen attack	Li *et al.* (2009) Zheng *et al.* (2013)
16	A_95_P170234	EH664856	APS reductase-like protein	3.58	Plant development and cold stress response	Phartiyal *et al.* (2008)
17	A_95_P259371	FG171287	APS reductase-like protein	3.57	Plant development and cold stress response	Phartiyal *et al.* (2008)
18	A_95_P316268	AY772945	Pectin methylesterase	3.45	Cell wall architecture and response to chilling stress	Qu *et al.* (2011)
19	A_95_P099313	BP534878	Methyltransferase-like protein	3.33	Response to abiotic/biotic stresses	Sahu *et al.* (2013)
20	A_95_P047176	BP131588	Ycf3 protein	3.14	PSI assembly and drought tolerance	Muchero *et al.* (2010)
21	A_95_P037263	BP128958	GST-like protein	3.06	Plant development and abiotic stress tolerance	Perez and Brown (2014)
22	A_95_P000116	FG157904	APS reductase-like protein	2.98	Plant development and cold stress response	Phartiyal *et al.* (2008)
23	A_95_P192712	EB432744	Calcium-binding protein	2.91	Response to environmental stresses	Tsou *et al.* (2012) Chen *et al.* (2015)
24	A_95_P173162	EH665543	Aspartate aminotransferase	2.81	Nitrogen metabolism and synthesis of amino acids	Zhou *et al.* (2009)
25	A_95_P221852	DV158128	Blue copper protein	2.68	Plant development and response to salinity and heavy metal stress	Ruan *et al.* (2011)
26	A_95_P173657	EH665660	Leucine-rich receptor-like kinase	2.56	Protein phosphorylation	Moscatiello *et al.* (2006)
27	A_95_P083300	BP528727	Ycf2 protein	2.54	PSI assembly and drought tolerance	Muchero *et al.* (2010)
28	A_95_P078860	BP527625	Receptor-like protein kinase	2.52	Response to abiotic stresses	Ye *et al.* (2017)
29	A_95_P128347	EB428011	Pectate lyase	2.52	Response to abiotic stresses	Palusa *et al.* (2007)
30	A_95_P122212	DW002241	Golgin subfamily protein	2.51	Golgi formation and membrane trafficking	Latijnhouwers *et al.* (2007)
31	A_95_P034973	BJ999201	RNA-binding protein	2.51	Tolerance to salt and drought stress	Ambrosone *et al.* (2015)
32	A_95_P297413	FG152847	ICR1-like protein	2.51	Plant growth	Li *et al.* (2008)
33	A_95_P035398	BP128462	Maturase K gene	2.46	Splicing of chloroplast group II introns	Vogel *et al.* (1999)
34	A_95_P157547	EH615593	Reticuline oxidase-like protein	2.41	Cell wall architecture and response to pathogen attack	Belchí-Navarro *et al.* (2013)
35	A_95_P159832	EH618298	CCR-like protein	2.40	Abiotic stress tolerance	Ghosh *et al.* (2014)
36	A_95_P190577	EH615701	LHT1-like protein	2.38	Ethylene responses	Shin *et al.* (2015)
37	A_95_P005336	BP130308	Pectin methylesterase inhibitor	2.36	Anti-fungal activity disease resistance and stress tolerance	An *et al.* (2008)
38	A_95_P053311	EB425896	Sulphur deficiency-induced protein	2.28	Utilization of stored sulfate	Howarth *et al.* (2009)
39	A_95_P018051	DV160720	SOD	2.27	Plant development and abiotic stress tolerance	Perez and Brown (2014)
40	A_95_P186302	EB436456	APS reductase-like protein	2.26	Plant development and cold stress response	Phartiyal *et al.* (2008)
41	A_95_P183277	DW001571	Putrescine *N*-methyltransferase	2.26	Biosynthesis of alkaloid and wound response	Sachan and Falcone (2002)
42	A_95_P155202	FG191506	Transcription initiation factor	2.26	Plant growth, development and abiotic stress tolerance	Singh *et al.* (2013)
43	A_95_P134087	EB435916	APS reductase-like protein	2.23	Plant development and cold stress response	Phartiyal *et al.* (2008)
44	A_95_P145077	EB448622	TRB-like protein	2.22	Telomere formation	Schrumpfová *et al.* (2014)
45	A_95_P164817	EH624155	Receptor-like protein kinase	2.22	Response to abiotic stresses	Ye *et al.* (2017)
46	A_95_P130417	EB430467	PPR-containing protein	2.19	Recovery of fertility	Bentolila *et al.* (2002)
47	A_95_P284013	FG139134	Receptor-like protein kinase	2.19	Response to abiotic stresses	Ye *et al.* (2017)
48	A_95_P258921	X06134	Nitrate reductase	2.19	Stress tolerance and plant growth	Zhang *et al.* (2014)
49	A_95_P131462	FG191106	PPIase-like protein	2.18	Salt stress response	Wang *et al.* (2014*b*)
50	A_95_P023626	AW032686	Chloroplast NAD(P)H dehydrogenase	2.17	Photosynthesis and growth under cold stress	Yamori *et al.* (2011)
51	A_95_P058111	BP134390	PPR-containing protein	2.15	Recovery of fertility	Bentolila *et al.* (2002)
52	A_95_P107022	CV018176	Sodium-coupled neutral amino acid transporter	2.15	Plant growth, development and salt stress response	Ortiz-Lopez *et al.* (2000) Popova *et al.* (2003)
53	A_95_P136752	EB680256	Granule-bound starch synthase	2.14	Amylose synthesis	Seung *et al.* (2015)
54	A_95_P221202	BP128310	GDSL esterase/lipase	2.12	Lipid metabolism, plant development, biotic and abiotic stress responses	Dong *et al.* (2016)
55	A_95_P248432	AM794263	Chloroplast ribosomal protein	2.09	Abiotic stress resistance	Liu *et al.* (2014)
56	A_95_P121002	DW000972	Glycine dehydrogenase	2.09	Photosynthesis and plant growth	Timm *et al.* (2012)
57	A_95_P249287	AF149251	Secretory peroxidase	2.08	Membrane protective function	Lüthje *et al.* (2011)
58	A_95_P134072	HO663864	APS reductase-like protein	2.08	Plant development and cold stress response	Phartiyal *et al.* (2008)
59	A_95_P161877	EH620463	UCH-like protein	2.08	Ubiquitin recycling and protein regulation	Isono and Nagel (2014)
60	A_95_P282903	AM847814	Tropinone reductase homolog	2.03	Biosynthesis of alkaloid	Kushwaha *et al.* (2013)
61	A_95_P315843	FG189231	Cyclin	2.01	Drought stress response	Zhou *et al.* (2013)

The WT and transgenic tobacco plants were grown in the greenhouse for 2 weeks and treated with 200 mM NaCl for 6 h; total RNA was extracted from seedlings to perform gene expression profiling by microarray analysis. Transcripts exhibiting more than 2-fold increase in *CbFAD3*-overexpressing seedlings compared with those of WT controls were considered to show significant changes. Values are means from three independent trials for transgenic and WT plants.

## Discussion

As a consequence of overexpressing a FAD3 gene (Zhang *et al.* 2005; Yu *et al.* 2009; Wang *et al.* 2014*a*), constitutively increased C18:3 content, accompanied by enhanced multiple-stress tolerance (Fig. 4), was observed in *CbFAD3*-transgenic tobacco plants. Furthermore, the high level of C18:3 resulting from *CbFAD3* overexpression in transgenic plants was approximately the same as that induced by drought stress in WT plants (Fig. 6A, B). Likewise, overexpression of *AtFAD7* leads to the same consequence in tobacco plants as low-temperature-induced C18:3 production, and confers low-temperature tolerance in young tobacco leaves (Kodama *et al.* 1995). These results indicate that overexpression of ω-3 FAD genes can constitutively increase C18:3 to the same level as that induced by abiotic stresses and thereby enhance the plant’s stress tolerance.

Previous studies have suggested that the enhanced stress tolerance is due to the modification of membrane fluidity caused by fatty acid desaturation, which prevents stress-induced membrane rigidification/disruption and maintains the structural and functional integrity of cell membranes (Mikami and Murata, 2003; Los and Murata, 2004; Zhang *et al.*, 2005; Upchurch, 2008; Yu, *et al.* 2009). Consistent with previous findings, fatty acid desaturation, in both transgenic and WT plants (Fig. 6A, B), helped the cell membranes to maintain optimal fluidity during abiotic stress (Fig. 6C). However, the distinct stress tolerance between these plants (Fig. 5) appears to weaken the dominant role of the maintenance of membrane fluidity in enhancing stress tolerance. More factors should, thus, be taken into consideration.

As an important shaper of Ca^2+^ signature in response to environmental stimuli, PM Ca^2+^-ATPase, which is primarily regulated at the post-translational level, plays a crucial role in stress signaling and adaptation (Beffagna *et al.*, 2005; Bose *et al.*, 2011; Shabala *et al.*, 2011). Interestingly, the experimental results along with correlation analysis showed that the activity of PM Ca^2+^-ATPase, in both *CbFAD3*-transgenic and WT plants, had a strong positive correlation with fatty acid unsaturation, especially with the level of C18:3, but was not correlated with membrane fluidity (Fig. 6A–C; Table 1). To the best of our knowledge, relevant studies have never been reported in plants, though there are findings reported from human and animal studies confirming that C18 unsaturated fatty acids increase the activity of PM Ca^2+^-ATPase in neutrophils (Hwang *et al.*, 2009), and membrane fluidity has no significant effect on the activity of sarcoplasmic reticulum Ca^2+^-ATPase (Madden *et al.*, 1981). The interpretation of the underlying mechanism is that certain unsaturated fatty acids may help form the active state of some membrane enzymes such as Ca^2+^-ATPase by penetrating into the protein core to displace the native interactions and destabilize the native state (Grossfield *et al.*, 2006; Hwang *et al.*, 2009).

Although the C18:3-induced activation of PM Ca^2+^-ATPase was found in both transgenic and WT plants (Fig. 6A, D; Table 1), the distinct stress tolerance between them suggests that the enhanced tolerance of transgenic plants might be associated with the sustained activation of PM Ca^2+^-ATPase induced by the constitutive accumulation of C18:3. Indeed, this assumption is strongly supported by a recent finding that overexpressing a PM Ca^2+^-ATPase gene (*OsACA6*) in tobacco plants, which is analogous to providing a sustained activation of PM Ca^2+^-ATPase in the whole plant, enhances the tolerance of plants to drought, salt, and cold stresses (Huda *et al.*, 2013*a*, 2014). As for the enhanced multiple-stress tolerance, Huda *et al.* (2013*a*) speculated that overexpression of *OsACA6* might enhance Ca^2+^ efflux and shape the [Ca^2+^]_cyt_ spike, thereby regulating the signaling mechanisms that promote ROS scavenging and the expression of stress-responsive genes. In our experiments, the enhanced Ca^2+^ efflux (Fig. 7A, B) and the lowered [Ca^2+^]_cyt_ elevation (Fig. 7C, D) resulting from the sustained activation of PM Ca^2+^-ATPase (Fig. 6D) were observed in *CbFAD3*-transgenic plants during the early stage of abiotic stresses. This confirms Huda’s speculation (Huda *et al.*, 2013*a*) along with the enhanced ROS scavenging (Fig. 8) and the increased expression of stress-responsive genes (Table 2). More importantly, all these results provide strong evidence for our assumption that the C18:3-induced sustained activation of PM Ca^2+^-ATPase, which regulated the stress-induced Ca^2+^ signaling through enhancing Ca^2+^ efflux and shaping of the [Ca^2+^]_cyt_ spike, is required for the multiple-stress tolerance of *CbFAD3*-overexpressing plants.

It is known that stress-induced [Ca^2+^]_cyt_ elevations vary in magnitude, frequency, and shape depending on the type and severity of stress and thus create a unique stress-specific Ca^2+^ signature that is subsequently decoded by signal transduction networks (Bose *et al.*, 2011). Given that the stress-induced [Ca^2+^]_cyt_ spikes in *CbFAD3*-transgenic plants were changed in magnitude and shape compared with those in WT controls (Fig. 7C, D), it is reasonable to assume that a proper regulation of the stress-specific Ca^2+^ signature might enhance plant stress tolerance through triggering distinct downstream responses. Although the exact criteria for the regulation are not clear, it needs at least to control the stress-induced [Ca^2+^]_cyt_ elevation within non-toxic levels (Dodd *et al.*, 2010; Huda *et al.*, 2013*a*), and retain the signaling role of the elevation (Case *et al.*, 2007; Huda *et al.*, 2013*b*).

Considering the cytotoxicity of the excess ROS resulting from the environmental stimulus, plant stress tolerance is often attributed to the enhanced ROS-scavenging ability (Gill and Tuteja, 2010; Huda *et al.*, 2013*a*). Wang *et al.* (2014*a*) found that overexpression of *LeFAD3* in tomato could enhance antioxidant enzyme activities and then salt stress tolerance. Similar phenomena (Figs 4, 8) were also found in *CbFAD3*-overexpressing tobacco plants. Correlation analysis showed that the antioxidant enzyme activities, especially the SOD activity, had a certain degree of positive correlation with the level of C18:3 and DBI (Table 1). A more than 2-fold increase in SOD mRNA was detected in transgenic plants compared with WT controls (Table 2; Supplementary Fig. S2), suggesting the existence of transcriptional or post-transcriptional regulation of SOD. Given that PM Ca^2+^-ATPase, which could be activated by C18:3 accumulation (Fig.6A, D; Table 1), is an important regulator in switching off the signal triggering ROS production (Beffagna *et al.*, 2005; Shabala *et al.*, 2011; Huda *et al.*, 2013*a*), at least a partial contribution of the PM Ca^2+^-ATPase to the activation of antioxidant enzymes cannot be ruled out. Additionally, because C18:3 plays a role in plant tolerance by serving as a sink of ROS (Mène-Saffrané *et al.*, 2009), *CbFAD3*-transgenic plants with the constitutive accumulation of C18:3 might possess more non-enzymatic antioxidant ability to counter the oxidative burst during the early stress response. It has been suggested that cross-tolerance, the enhanced ability of a plant to tolerate multiple stresses, results partly from the overlap between the ROS signaling mechanisms (Perez and Brown, 2014). Therefore, the multiple-stress tolerance of *CbFAD3*-overexpressing plants was associated with enzymatic and non-enzymatic ROS management brought about by the constitutively increased C18:3 content as well as by the sustained activation of PM Ca^2+^-ATPase.

A recent study confirmed that exogenous C18:3 can modulate the expression of stress-responsive genes, especially mediated by ROS (Mata-Pérez *et al.*, 2015). This prompts speculation that the constitutive accumulation of C18:3 may also affect the increased expression of stress-responsive genes in *CbFAD3*-overexpressing plants (Table 2). Microarray analysis showed that the highest increased expression (15.02-fold) belonged to the gene for cytochrome *c* oxidase, a respiration- and ROS-related protein, which is controlled by the C18:1 and C18:3 content of membranes, and has a critical role in chilling and salt tolerance (De Santis *et al.*, 1999; Yan *et al.*, 2005). Among the other genes, the genes for protein kinases, ROS-responsive proteins, transcription factors, signaling proteins, disease resistance proteins, chromatin remodeling proteins, and DNA methylation proteins, which are induced by Ca^2+^ or ROS signaling and in turn affect the signal pathway and gene expression, play a key role in stress tolerance (Moscatiello *et al.*, 2006; Xu *et al.*, 2009; Kim *et al.*, 2010; Zhang *et al.*, 2012; Aghaei and Komatsu, 2013; Sahu *et al.*, 2013; Perez and Brown, 2014; Yang *et al.*, 2014; Cao *et al.*, 2015). Moreover, nitrogen/sulfur metabolism-related proteins and alkaloid biosynthesis-related proteins are confirmed to participate in the stress response for generating diverse physiologically active substances (Sachan and Falcone, 2002; Wang *et al.*, 2005; Phartiyal *et al.*, 2008; Aghaei and Komatsu, 2013), and the increased expression of their genes was detected. Notably, the increased transcript accumulation of cell wall/membrane-related proteins and photosynthesis-related proteins provides a rational explanation for maintaining membrane and photosynthetic status in transgenic plants under abiotic stresses (Fig. 5). Also, the data showed an increased mRNA level of cell cycle-related proteins, which can regulate the drought stress response by inhibiting ROS accumulation (Zhou *et al.*, 2013). These results demonstrate that the multiple-stress tolerance of *CbFAD3*-overexpressing plants was related to the increased expression of stress-responsive genes, most of which not only are coordinated with one other, but are affected by C18:3 or the C18:3-induced regulation of Ca^2+^ and ROS.

**Fig. 9.**
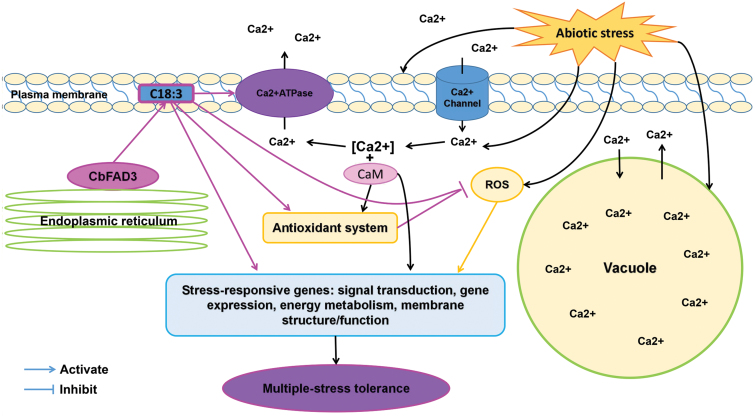
Hypothetical model for the role of *CbFAD3* in plant stress adaptation. Abiotic stress causes an increase in [Ca^2+^]_cyt_ and ROS. Overexpression of *CbFAD3* constitutively increases the level of C18:3, which maintains membrane fluidity, and alters the stress-induced Ca^2+^ signaling through sustainably activating PM Ca^2+^-ATPase. The constitutively increased C18:3 along with the changed [Ca^2+^]_cyt_ also switches off ROS production by activating the antioxidant defense system, and then increases the expression of various stress-responsive genes, resulting in stress responses. Overall, the *CbFAD3*-triggered integrated regulation of membrane, Ca^2+^, ROS, and stress-responsive genes contributes to plant multiple-stress tolerance.

In conclusion, *CbFAD3* is a *C. bungeana* ω-3 FAD gene involved in stress adaptation. Overexpression of *CbFAD3* in tobacco enhanced multiple-stress tolerance through C18:3-induced integrated regulation, including membrane stabilization, [Ca^2+^]_cyt_ modification, ROS management, and the increased expression of stress-responsive genes (Fig. 9). These results reveal a comprehensive mechanism for the involvement of *CbFAD3* in response to environmental stresses and should provide a potential target for crop improvement.

## Supplementary data

Supplementary data are available at *JXB* online.

Fig. S1. Molecular analysis of *CbFAD3*-overexpressing tobacco plants.

Fig. S2. qPCR analysis for the increased expression of six genes in *CbFAD3*-overexpressing tobacco plants under salt treatment.

Table S1. Information for primers used for gene cloning and vector construction.

Table S2. The accession number of ω-3 FADs included in alignment.

Table S3. Information for primers used for qRT-PCR.

Table S4. Fatty acid composition of *S. cerevesiae* cells overexpressing *CbFAD3* grown at 20 °C.

Supplementary figures and Tables

## References

[CIT0001] Aghaei K, Komatsu S 2013 Crop and medicinal plants proteomics in response to salt stress. Frontiers in Plant Science 4, 8. 23386857 10.3389/fpls.2013.00008 PMC3560237

[CIT0002] Ambrosone A, Batelli G, Nurcato R et al 2015 The Arabidopsis RNA-binding protein AtRGGA regulates tolerance to salt and drought stress. Plant Physiology 168, 292–306. 25783413 10.1104/pp.114.255802 PMC4424017

[CIT0003] An SH, Sohn KH, Choi HW et al 2008 Pepper pectin methylesterase inhibitor protein CaPMEI1 is required for antifungal activity, basal disease resistance and abiotic stress tolerance. Planta 228, 61–78. 18327607 10.1007/s00425-008-0719-z PMC2413075

[CIT0004] Baxter A, Mittler R, Suzuki N 2014 ROS as key players in plant stress signalling. Journal of Experimental Botany 65, 1229–1240. 24253197 10.1093/jxb/ert375

[CIT0005] Beffagna N, Buffoli B, Busi C 2005 Modulation of reactive oxygen species production during osmotic stress in *Arabidopsis thaliana* cultured cells: involvement of the plasma membrane Ca^2+^-ATPase and H^+^-ATPase. Plant & Cell Physiology 46, 1326–1339. 15937326 10.1093/pcp/pci142

[CIT0006] Belchí-Navarro S, Almagro L, Sabater-Jara AB, Fernández-Pérez F, Bru R, Pedreño MA 2013 Induction of *trans*-resveratrol and extracellular pathogenesis-related proteins in elicited suspension cultured cells of *Vitis vinifera* cv Monastrell. Journal of Plant Physiology 170, 258–264. 23127362 10.1016/j.jplph.2012.10.003

[CIT0007] Bentolila S, Alfonso AA, Hanson MR 2002 A pentatricopeptide repeat-containing gene restores fertility to cytoplasmic male-sterile plants. Proceedings of the National Academy of Sciences, USA 99, 10887–10892. 10.1073/pnas.102301599 PMC125068 12136123

[CIT0008] Berberich T, Harada M, Sugawara K, Kodama H, Iba K, Kusano T 1998 Two maize genes encoding ω-3 fatty acid desaturase and their differential expression to temperature. Plant Molecular Biology 36, 297–306. 9484441 10.1023/a:1005993408270

[CIT0009] Bose J, Pottosin II, Shabala SS, Palmgren MG, Shabala S 2011 Calcium efflux systems in stress signaling and adaptation in plants. Frontiers in Plant Science 2, 85. 22639615 10.3389/fpls.2011.00085 PMC3355617

[CIT0010] Bradford MM 1976 A rapid and sensitive method for the quantitation of microgram quantities of protein utilizing the principle of protein-dye binding. Analytical Biochemistry 72, 248–254. 942051 10.1016/0003-2697(76)90527-3

[CIT0011] Cao YR, Chen HW, Li ZG, Tao JJ, Ma B, Zhang WK, Chen SY, Zhang JS 2015 Tobacco ankyrin protein NEIP2 interacts with ethylene receptor NTHK1 and regulates plant growth and stress responses. Plant & Cell Physiology 56, 803–818. 25634961 10.1093/pcp/pcv009

[CIT0012] Case RM, Eisner D, Gurney A, Jones O, Muallem S, Verkhratsky A 2007 Evolution of calcium homeostasis: from birth of the first cell to an omnipresent signalling system. Cell Calcium 42, 345–350. 17574670 10.1016/j.ceca.2007.05.001

[CIT0013] Chen C, Sun X, Duanmu H et al 2015 *GsCML27*, a gene encoding a calcium-binding Ef-hand protein from *Glycine soja*, plays differential roles in plant responses to bicarbonate, salt and osmotic stresses. PLoS One 10, 141888. 10.1371/journal.pone.0141888 PMC4638360 26550992

[CIT0014] De Santis A, Landi P, Genchi G 1999 Changes of mitochondrial properties in maize seedlings associated with selection for germination at low temperature. Fatty acid composition, cytochrome *c* oxidase, and adenine nucleotide translocase activities. Plant Physiology 119, 743–754. 9952471 10.1104/pp.119.2.743 PMC32152

[CIT0015] Di C, Li M, Long F, Bai M, Liu Y, Zheng X, Xu S, Xiang Y, Sun Z, An L 2009 Molecular cloning, functional analysis and localization of a novel gene encoding polygalacturonase-inhibiting protein in *Chorispora bungeana*. Planta 231, 169–178. 19885675 10.1007/s00425-009-1039-7

[CIT0016] Dodd AN, Kudla J, Sanders D 2010 The language of calcium signaling. Annual Review of Plant Biology 61, 593–620. 10.1146/annurev-arplant-070109-104628 20192754

[CIT0017] Dong X, Yi H, Han CT, Nou IS, Hur Y 2016 GDSL esterase/lipase genes in *Brassica rapa* L.: genome-wide identification and expression analysis. Molecular Genetics and Genomics 291, 531–542. 26423069 10.1007/s00438-015-1123-6

[CIT0018] Dyer JM, Chapital DC, Cary JW, Pepperman AB 2001 Chilling-sensitive, post-transcriptional regulation of a plant fatty acid desaturase expressed in yeast. Biochemical and Biophysical Research Communications 282, 1019–1025. 11352654 10.1006/bbrc.2001.4667

[CIT0019] Fu XY, Chang JF, An LZ et al 2006 Association of the cold-hardiness of *Chorispora bungeana* with the distribution and accumulation of calcium in the cells and tissues. Environmental and Experimental Botany 55, 282–293.

[CIT0020] Gangadhar BH, Sajeesh K, Venkatesh J et al 2016 Enhanced tolerance of transgenic potato plants over-expressing non-specific lipid transfer protein-1 (StnsLTP1) against multiple abiotic stresses. Frontiers in Plant Science 7, 1228. 27597854 10.3389/fpls.2016.01228 PMC4993012

[CIT0021] Garcés R, Mancha M 1993 One-step lipid extraction and fatty acid methyl esters preparation from fresh plant tissues. Analytical Biochemistry 211, 139–143. 8323025 10.1006/abio.1993.1244

[CIT0022] Ghosh R, Choi B, Cho BK, Lim HS, Park SU, Bae HJ, Natarajan S, Bae H 2014 Characterization of developmental- and stress-mediated expression of cinnamoyl-CoA reductase in kenaf (*Hibiscus cannabinus* L.). The Scientific World Journal 2014, 601845. 24723816 10.1155/2014/601845 PMC3958759

[CIT0023] Giacometti S, Marrano CA, Bonza MC, Luoni L, Limonta M, De Michelis MI 2012 Phosphorylation of serine residues in the N-terminus modulates the activity of ACA8, a plasma membrane Ca^2+^-ATPase of *Arabidopsis thaliana*. Journal of Experimental Botany 63, 1215–1224. 22090438 10.1093/jxb/err346 PMC3276087

[CIT0024] Gill SS, Tuteja N 2010 Reactive oxygen species and antioxidant machinery in abiotic stress tolerance in crop plants. Plant Physiology and Biochemistry 48, 909–930. 20870416 10.1016/j.plaphy.2010.08.016

[CIT0025] Grossfield A, Feller SE, Pitman MC 2006 A role for direct interactions in the modulation of rhodopsin by ω-3 polyunsaturated lipids. Proceedings of the National Academy of Sciences, USA 103, 4888–4893. 10.1073/pnas.0508352103 PMC1458765 16547139

[CIT0026] Haruta M, Sussman MR 2012 The effect of a genetically reduced plasma membrane protonmotive force on vegetative growth of Arabidopsis. Plant Physiology 158, 1158–1171. 22214817 10.1104/pp.111.189167 PMC3291248

[CIT0027] Horsch RB 1985 A simple and general method for transferring genes into plants. Science 227, 1229–1231. 17757866 10.1126/science.227.4691.1229

[CIT0028] Horvath A, Riezman H 1994 Rapid protein extraction from *Saccharomyces cerevisiae*. Yeast 10, 1305–1310. 7900419 10.1002/yea.320101007

[CIT0029] Howarth JR, Parmar S, Barraclough PB, Hawkesford MJ 2009 A sulphur deficiency-induced gene, *sdi1*, involved in the utilization of stored sulphate pools under sulphur-limiting conditions has potential as a diagnostic indicator of sulphur nutritional status. Plant Biotechnology Journal 7, 200–209. 19154231 10.1111/j.1467-7652.2008.00391.x

[CIT0030] Huda KM, Banu MS, Garg B, Tula S, Tuteja R, Tuteja N 2013 *a* OsACA6, a P-type IIB Ca²⁺ATPase promotes salinity and drought stress tolerance in tobacco by ROS scavenging and enhancing the expression of stress-responsive genes. The Plant Journal 76, 997–1015. 24128296 10.1111/tpj.12352

[CIT0031] Huda KM, Banu MS, Yadav S et al 2014 Salinity and drought tolerant OsACA6 enhances cold tolerance in transgenic tobacco by interacting with stress-inducible proteins. Plant Physiology and Biochemistry 82, 229–238. 24992889 10.1016/j.plaphy.2014.06.007

[CIT0032] Huda KM, Yadav S, Akhter Banu MS, Trivedi DK, Tuteja N 2013 *b* Genome-wide analysis of plant-type II Ca^2+^ATPases gene family from rice and Arabidopsis: potential role in abiotic stresses. Plant Physiology and Biochemistry 65, 32–47. 23416494 10.1016/j.plaphy.2013.01.002

[CIT0033] Hwang TL, Su YC, Chang HL, Leu YL, Chung PJ, Kuo LM, Chang YJ 2009 Suppression of superoxide anion and elastase release by C18 unsaturated fatty acids in human neutrophils. Journal of Lipid Research 50, 1395–1408. 19295184 10.1194/jlr.M800574-JLR200 PMC2694338

[CIT0034] Im YJ, Han O, Chung GC, Cho BH 2002 Antisense expression of an Arabidopsis omega-3 fatty acid desaturase gene reduces salt/drought tolerance in transgenic tobacco plants. Molecules and Cells 13, 264–271. 12018849

[CIT0035] Isono E, Nagel MK 2014 Deubiquitylating enzymes and their emerging role in plant biology. Frontiers in Plant Science 5, 56. 24600466 10.3389/fpls.2014.00056 PMC3928566

[CIT0036] Kim JM, To TK, Nishioka T, Seki M 2010 Chromatin regulation functions in plant abiotic stress responses. Plant, Cell & Environment 33, 604–611. 10.1111/j.1365-3040.2009.02076.x 19930132

[CIT0037] Kodama H, Akagi H, Kusumi K, Fujimura T, Iba K 1997 Structure, chromosomal location and expression of a rice gene encoding the microsome ω-3 fatty acid desaturase. Plant Molecular Biology 33, 493–502. 9049269 10.1023/a:1005726210977

[CIT0038] Kodama H, Horiguchi G, Nishiuchi T, Nishimura M, Iba K 1995 Fatty acid desaturation during chilling acclimation is one of the factors involved in conferring low-temperature tolerance to young tobacco leaves. Plant Physiology 107, 1177–1185. 12228424 10.1104/pp.107.4.1177 PMC157250

[CIT0039] Kumar R, Tran LS, Neelakandan AK, Nguyen HT 2012 Higher plant cytochrome b5 polypeptides modulate fatty acid desaturation. PLoS One 7, 31370. 10.1371/journal.pone.0031370 PMC3285619 22384013

[CIT0040] Kushwaha AK, Sangwan NS, Trivedi PK, Negi AS, Misra L, Sangwan RS 2013 Tropine forming tropinone reductase gene from *Withania somnifera* (Ashwagandha): biochemical characteristics of the recombinant enzyme and novel physiological overtones of tissue-wide gene expression patterns. PLoS One 8, e74777. 24086372 10.1371/journal.pone.0074777 PMC3783447

[CIT0041] Latijnhouwers M, Gillespie T, Boevink P, Kriechbaumer V, Hawes C, Carvalho CM 2007 Localization and domain characterization of Arabidopsis golgin candidates. Journal of Experimental Botany 58, 4373–4386. 18182439 10.1093/jxb/erm304

[CIT0042] Li H, Wang Y, Jiang J, Liu G, Gao C, Yang C 2009 Identification of genes responsive to salt stress on *Tamarix hispida* roots. Gene 433, 65–71. 19146931 10.1016/j.gene.2008.12.007

[CIT0043] Li S, Gu Y, Yan A, Lord E, Yang ZB 2008 RIP1 (ROP Interactive Partner 1)/ICR1 marks pollen germination sites and may act in the ROP1 pathway in the control of polarized pollen growth. Molecular Plant 1, 1021–1035. 19825600 10.1093/mp/ssn051 PMC9345201

[CIT0044] Liu XD, Xie L, Wei Y, Zhou X, Jia B, Liu J, Zhang S 2014 Abiotic stress resistance, a novel moonlighting function of ribosomal protein RPL44 in the halophilic fungus *Aspergillus glaucus*. Applied and Environmental Microbiology 80, 4294–4300. 24814782 10.1128/AEM.00292-14 PMC4068663

[CIT0045] Los DA, Murata N 1998 Structure and expression of fatty acid desaturases. Biochimica et Biophysica Acta 1394, 3–15. 9767077 10.1016/s0005-2760(98)00091-5

[CIT0046] Los DA, Murata N 2004 Membrane fluidity and its roles in the perception of environmental signals. Biochimica et Biophysica Acta-Biomembranes 1666, 142–157. 10.1016/j.bbamem.2004.08.002 15519313

[CIT0047] Loutre C, Wicker T, Travella S et al 2009 Two different CC-NBS-LRR genes are required for *Lr10*-mediated leaf rust resistance in tetraploid and hexaploid wheat. The Plant Journal 60, 1043–1054. 19769576 10.1111/j.1365-313X.2009.04024.x

[CIT0048] Lüthje S, Meisrimler CN, Hopff D, Möller B 2011 Phylogeny, topology, structure and functions of membrane-bound class III peroxidases in vascular plants. Phytochemistry 72, 1124–1135. 21211808 10.1016/j.phytochem.2010.11.023

[CIT0049] Madden TD, King MD, Quinn PJ 1981 The modulation of Ca^2+^-ATPase activity of sarcoplasmic reticulum by membrane cholesterol. The effect of enzyme coupling. Biochimica et Biophysica Acta 641, 265–269. 6452166 10.1016/0005-2736(81)90590-3

[CIT0050] Mata-Pérez C, Sánchez-Calvo B, Begara-Morales JC et al 2015 Transcriptomic profiling of linolenic acid-responsive genes in ROS signaling from RNA-seq data in Arabidopsis. Frontiers in Plant Science 6, 122. 25852698 10.3389/fpls.2015.00122 PMC4362301

[CIT0051] Mène-Saffrané L, Dubugnon L, Chételat A, Stolz S, Gouhier-Darimont C, Farmer EE 2009 Nonenzymatic oxidation of trienoic fatty acids contributes to reactive oxygen species management in Arabidopsis. The Journal of Biological Chemistry 284, 1702–1708. 18996838 10.1074/jbc.M807114200

[CIT0052] Miao BH, Han XG, Zhang WH 2010 The ameliorative effect of silicon on soybean seedlings grown in potassium-deficient medium. Annals of Botany 105, 967–973. 20338952 10.1093/aob/mcq063 PMC2876006

[CIT0053] Mikami K, Murata N 2003 Membrane fluidity and the perception of environmental signals in cyanobacteria and plants. Progress in Lipid Research 42, 527–543. 14559070 10.1016/s0163-7827(03)00036-5

[CIT0054] Moscatiello R, Mariani P, Sanders D, Maathuis FJ 2006 Transcriptional analysis of calcium-dependent and calcium-independent signalling pathways induced by oligogalacturonides. Journal of Experimental Botany 57, 2847–2865. 16868046 10.1093/jxb/erl043

[CIT0055] Muchero W, Ehlers JD, Roberts PA 2010 Restriction site polymorphism-based candidate gene mapping for seedling drought tolerance in cowpea [*Vigna unguiculata* (L.) Walp.]. Theoretical and Applied Genetics 120, 509–518. 19834655 10.1007/s00122-009-1171-6 PMC2807941

[CIT0056] Mykytczuk NCS, Trevorsb JT, Leduca LG, Ferronic GD 2007 Fluorescence polarization in studies of bacterial cytoplasmic membrane fluidity under environmental stress. Progress in Biophysics and Molecular Biology 95, 60–82. 17628643 10.1016/j.pbiomolbio.2007.05.001

[CIT0057] Palmgren MG 2001 Plant plasma membrane H^+^-ATPases: powerhouses for nutrient uptake. Annual Review of Plant Physiology and Plant Molecular Biology 52, 817–845. 10.1146/annurev.arplant.52.1.817 11337417

[CIT0058] Palusa SG, Golovkin M, Shin SB, Richardson DN, Reddy AS 2007 Organ-specific, developmental, hormonal and stress regulation of expression of putative pectate lyase genes in Arabidopsis. New Phytologist 174, 537–550. 17447910 10.1111/j.1469-8137.2007.02033.x

[CIT0059] Perez IB, Brown PJ 2014 The role of ROS signaling in cross-tolerance: from model to crop. Frontiers in Plant Science 5, 754. 25566313 10.3389/fpls.2014.00754 PMC4274871

[CIT0060] Pfaffl MW 2001 A new mathematical model for relative quantification in real-time RT-PCR. Nucleic Acids Research 29, e45. 11328886 10.1093/nar/29.9.e45 PMC55695

[CIT0061] Phartiyal P, Kim WS, Cahoon RE, Jez JM, Krishnan HB 2008 The role of 5′-adenylylsulfate reductase in the sulfur assimilation pathway of soybean: molecular cloning, kinetic characterization, and gene expression. Phytochemistry 69, 356–364. 17761201 10.1016/j.phytochem.2007.07.013

[CIT0062] Popova OV, Dietz KJ, Golldack D 2003 Salt-dependent expression of a nitrate transporter and two amino acid transporter genes in *Mesembryanthemum crystallinum*. Plant Molecular Biology 52, 569–578. 12956527 10.1023/a:1024802101057

[CIT0063] Ortiz-Lopez A, Chang H, Bush DR 2000 Amino acid transporters in plants. Biochimica et Biophysica Acta 1465, 275–280. 10748260 10.1016/s0005-2736(00)00144-9

[CIT0064] Qu T, Liu R, Wang W, An L, Chen T, Liu G, Zhao Z 2011 Brassinosteroids regulate pectin methylesterase activity and AtPME41 expression in Arabidopsis under chilling stress. Cryobiology 63, 111–117. 21819976 10.1016/j.cryobiol.2011.07.003

[CIT0065] Román Á, Andreu V, Hernández ML, Lagunas B, Picorel R, Martínez-Rivas JM, Alfonso M 2012 Contribution of the different omega-3 fatty acid desaturase genes to the cold response in soybean. Journal of Experimental Botany 63, 4973–4982. 22865909 10.1093/jxb/ers174 PMC3427996

[CIT0066] Román Á, Hernández ML, Soria-García Á, López-Gomollón S, Lagunas B, Picorel R, Martínez-Rivas JM, Alfonso M 2015 Non-redundant contribution of the plastidial FAD8 ω-3 desaturase to glycerolipid unsaturation at different temperatures in Arabidopsis. Molecular Plant 8, 1599–1611. 26079601 10.1016/j.molp.2015.06.004

[CIT0067] Ruan XM, Luo F, Li DD, Zhang J, Liu ZH, Xu WL, Huang GQ, Li XB 2011 Cotton BCP genes encoding putative blue copper-binding proteins are functionally expressed in fiber development and involved in response to high-salinity and heavy metal stresses. Physiologia Plantarum 141, 71–83. 21029107 10.1111/j.1399-3054.2010.01420.x

[CIT0068] Rutschow HL, Baskin TI, Kramer EM 2014 The carrier AUXIN RESISTANT (AUX1) dominates auxin flux into Arabidopsis protoplasts. New Phytologist 204, 536–544. 25039492 10.1111/nph.12933

[CIT0069] Sachan N, Falcone DL 2002 Wound-induced gene expression of putrescine *N*-methyltransferase in leaves of *Nicotiana tabacum*. Phytochemistry 61, 797–805. 12453572 10.1016/s0031-9422(02)00427-2

[CIT0070] Sahu PP, Pandey G, Sharma N, Puranik S, Muthamilarasan M, Prasad M 2013 Epigenetic mechanisms of plant stress responses and adaptation. Plant Cell Reports 32, 1151–1159. 23719757 10.1007/s00299-013-1462-x

[CIT0071] Schmidt GW, Delaney SK 2010 Stable internal reference genes for normalization of real-time RT-PCR in tobacco (*Nicotiana tabacum*) during development and abiotic stress. Molecular Genetics and Genomics 283, 233–241. 20098998 10.1007/s00438-010-0511-1

[CIT0072] Schrumpfová PP, Vychodilová I, Dvořáčková M, Majerská J, Dokládal L, Schořová S, Fajkus J 2014 Telomere repeat binding proteins are functional components of Arabidopsis telomeres and interact with telomerase. The Plant Journal 77, 770–781. 24397874 10.1111/tpj.12428 PMC4282523

[CIT0073] Sedbrook JC, Carroll KL, Hung KF, Masson PH, Somerville CR 2002 The Arabidopsis *SKU5* gene encodes an extracellular glycosyl phosphatidylinositol-anchored glycoprotein involved in directional root growth. The Plant Cell 14, 1635–1648. 12119380 10.1105/tpc.002360 PMC150712

[CIT0074] Senthil-Kumar M, Hema R, Suryachandra TR, Ramegowda HV, Gopalakrishna R, Rama N, Udayakumar M, Mysore KS 2010 Functional characterization of three water deficit stress-induced genes in tobacco and Arabidopsis: an approach based on gene down regulation. Plant Physiology and Biochemistry 48, 35–44. 19811926 10.1016/j.plaphy.2009.09.005

[CIT0075] Seung D, Soyk S, Coiro M, Maier BA, Eicke S, Zeeman SC 2015 PROTEIN TARGETING TO STARCH is required for localising GRANULE-BOUND STARCH SYNTHASE to starch granules and for normal amylose synthesis in Arabidopsis. PLoS Biology 13, e1002080. 25710501 10.1371/journal.pbio.1002080 PMC4339375

[CIT0076] Shabala S, Baekgaard L, Shabala L, Fuglsang A, Babourina O, Palmgren MG, Cuin TA, Rengel Z, Nemchinov LG 2011 Plasma membrane Ca²^+^ transporters mediate virus-induced acquired resistance to oxidative stress. Plant, Cell & Environment 34, 406–417. 10.1111/j.1365-3040.2010.02251.x 21062316

[CIT0077] Shi Y, An L, Zhang M, Huang C, Zhang H, Xu S 2008 Regulation of the plasma membrane during exposure to low temperatures in suspension-cultured cells from a cryophyte (*Chorispora bungeana*). Protoplasma 232, 173–181. 18421547 10.1007/s00709-008-0291-1

[CIT0078] Shin K, Lee S, Song WY et al 2015 Genetic identification of ACC-RESISTANT2 reveals involvement of LYSINE HISTIDINE TRANSPORTER1 in the uptake of 1-aminocyclopropane-1-carboxylic acid in *Arabidopsis thaliana*. Plant & Cell Physiology 56, 572–582. 25520403 10.1093/pcp/pcu201

[CIT0079] Singh B, Chauhan H, Khurana JP, Khurana P, Singh P 2013 Evidence for the role of wheat eukaryotic translation initiation factor 3 subunit g (TaeIF3g) in abiotic stress tolerance. Gene 532, 177–185. 24084365 10.1016/j.gene.2013.09.078

[CIT0080] Sun M, Jia B, Cui N, Wen Y, Duanmu H, Yu Q, Xiao J, Sun X, Zhu Y 2016 Functional characterization of a *Glycine soja* Ca^2+^ATPase in salt-alkaline stress responses. Plant Molecular Biology 90, 419–434. 26801329 10.1007/s11103-015-0426-7

[CIT0081] Timm S, Florian A, Arrivault S, Stitt M, Fernie AR, Bauwe H 2012 Glycine decarboxylase controls photosynthesis and plant growth. FEBS Letters 586, 3692–3697. 22982108 10.1016/j.febslet.2012.08.027

[CIT0082] Tsou PL, Lee SY, Allen NS, Winter-Sederoff H, Robertson D 2012 An ER-targeted calcium-binding peptide confers salt and drought tolerance mediated by CIPK6 in *Arabidopsis*. Planta 235, 539–552. 21971994 10.1007/s00425-011-1522-9

[CIT0083] Upchurch RG 2008 Fatty acid unsaturation, mobilization, and regulation in the response of plants to stress. Biotechnology Letters 30, 967–977. 18227974 10.1007/s10529-008-9639-z

[CIT0084] Vogel J, Börner T, Hess WR 1999 Comparative analysis of splicing of the complete set of chloroplast group II introns in three higher plant mutants. Nucleic Acids Research 27, 3866–3874. 10481026 10.1093/nar/27.19.3866 PMC148650

[CIT0085] Wang H, Liu D, Sun J, Zhang A 2005 Asparagine synthetase gene *TaASN1* from wheat is up-regulated by salt stress, osmotic stress and ABA. Journal of Plant Physiology 162, 81–89. 15700423 10.1016/j.jplph.2004.07.006

[CIT0086] Wang HS, Yu C, Tang XF, Zhu ZJ, Ma NN, Meng QW 2014 *a* A tomato endoplasmic reticulum (ER)-type omega-3 fatty acid desaturase (LeFAD3) functions in early seedling tolerance to salinity stress. Plant Cell Reports 33, 131–142. 24129846 10.1007/s00299-013-1517-z

[CIT0087] Wang P, Li XZ, Cui HR, Feng YG, Wang XY 2014 *b* Identification and functional analysis of a novel parvulin-type peptidyl-prolyl isomerase from *Gossypium hirsutum*. Plant Physiology and Biochemistry 76, 58–66. 24468661 10.1016/j.plaphy.2013.12.020

[CIT0088] Widell S, Larsson C 1990 A critical evaluation of markers used in plasma membrane purification. In: Larsson C, Møller IM, eds. The plant plasma membrane. Berlin, Heidelberg: Springer, 16–43.

[CIT0089] Wu J, Qu T, Chen S, Zhao Z, An L 2009 Molecular cloning and characterization of a gamma-glutamylcysteine synthetase gene from *Chorispora bungeana*. Protoplasma 235, 27–36. 19082776 10.1007/s00709-008-0026-3

[CIT0090] Xu ZS, Liu L, Ni ZY, Liu P, Chen M, Li LC, Chen YF, Ma YZ 2009 W55a encodes a novel protein kinase that is involved in multiple stress responses. Journal of Integrative Plant Biology 51, 58–66. 19166495 10.1111/j.1744-7909.2008.00776.x

[CIT0091] Yamori W, Sakata N, Suzuki Y, Shikanai T, Makino A 2011 Cyclic electron flow around photosystem I via chloroplast NAD(P)H dehydrogenase (NDH) complex performs a significant physiological role during photosynthesis and plant growth at low temperature in rice. The Plant Journal 68, 966–976. 21848656 10.1111/j.1365-313X.2011.04747.x

[CIT0092] Yan S, Tang Z, Su W, Sun W 2005 Proteomic analysis of salt stress-responsive proteins in rice root. Proteomics 5, 235–244. 15672456 10.1002/pmic.200400853

[CIT0093] Yang A, Dai X, Zhang WH 2012 A R2R3-type MYB gene, *OsMYB2*, is involved in salt, cold, and dehydration tolerance in rice. Journal of Experimental Botany 63, 2541–2556. 22301384 10.1093/jxb/err431 PMC3346221

[CIT0094] Yang Y, Ma C, Xu Y et al 2014 A zinc finger protein regulates flowering time and abiotic stress tolerance in chrysanthemum by modulating gibberellin biosynthesis. The Plant Cell 26, 2038–2054. 24858937 10.1105/tpc.114.124867 PMC4079367

[CIT0095] Ye Y, Ding Y, Jiang Q, Wang F, Sun J, Zhu C 2017 The role of receptor-like protein kinases (RLKs) in abiotic stress response in plants. Plant Cell Reports 36, 235–242. 27933379 10.1007/s00299-016-2084-x

[CIT0096] Yu C, Wang HS, Yang S, Tang XF, Duan M, Meng QW 2009 Overexpression of endoplasmic reticulum omega-3 fatty acid desaturase gene improves chilling tolerance in tomato. Plant Physiology and Biochemistry 47, 1102–1112. 19648018 10.1016/j.plaphy.2009.07.008

[CIT0097] Zhang GB, Yi HY, Gong JM 2014 The Arabidopsis ethylene/jasmonic acid-NRT signaling module coordinates nitrate reallocation and the trade-off between growth and environmental adaptation. The Plant Cell 26, 3984–3998. 25326291 10.1105/tpc.114.129296 PMC4247569

[CIT0098] Zhang L, Si J, Zeng F, An L 2009 Molecular cloning and characterization of a ferritin gene upregulated by cold stress in *Chorispora bungeana*. Biological Trace Element Research 128, 269–283. 19034392 10.1007/s12011-008-8275-8

[CIT0099] Zhang L, Zhao G, Jia J, Liu X, Kong X 2012 Molecular characterization of 60 isolated wheat MYB genes and analysis of their expression during abiotic stress. Journal of Experimental Botany 63, 203–214. 21934119 10.1093/jxb/err264 PMC3245462

[CIT0100] Zhang M, Barg R, Yin M, Gueta-Dahan Y, Leikin-Frenkel A, Salts Y, Shabtai S, Ben-Hayyim G 2005 Modulated fatty acid desaturation via overexpression of two distinct ω-3 desaturases differentially alters tolerance to various abiotic stresses in transgenic tobacco cells and plants. The Plant Journal 44, 361–371. 16236147 10.1111/j.1365-313X.2005.02536.x

[CIT0101] Zhang T, Liu Y, Xue L, Xu S, Chen T, Yang T, Zhang L, An L 2006 Molecular cloning and characterization of a novel MAP kinase gene in *Chorispora bungeana*. Plant Physiology and Biochemistry 44, 78–84. 16531060 10.1016/j.plaphy.2006.01.001

[CIT0102] Zhang YM, Wang CC, Hu HH, Yang L 2011 Cloning and expression of three fatty acid desaturase genes from cold-sensitive lima bean (*Phaseolus lunatus* L.). Biotechnology Letters 33, 395–401. 20953666 10.1007/s10529-010-0432-4

[CIT0103] Zhao Z, Tan L, Dang C, Zhang H, Wu Q, An L 2012 Deep-sequencing transcriptome analysis of chilling tolerance mechanisms of a subnival alpine plant, *Chorispora bungeana*. BMC Plant Biology 12, 222. 23171377 10.1186/1471-2229-12-222 PMC3571968

[CIT0104] Zheng W, Ma L, Zhao J, Li Z, Sun F, Lu X 2013 Comparative transcriptome analysis of two rice varieties in response to rice stripe virus and small brown planthoppers during early interaction. PLoS One 8, e82126. 24358146 10.1371/journal.pone.0082126 PMC3864904

[CIT0105] Zhou XF, Jin YH, Yoo CY, Lin XL, Kim WY, Yun DJ, Bressan RA, Hasegawa PM, Jin JB 2013 CYCLIN H;1 regulates drought stress responses and blue light-induced stomatal opening by inhibiting reactive oxygen species accumulation in Arabidopsis. Plant Physiology 162, 1030–1041. 23656895 10.1104/pp.113.215798 PMC3668038

[CIT0106] Zhou Y, Cai H, Xiao J, Li X, Zhang Q, Lian X 2009 Over-expression of aspartate aminotransferase genes in rice resulted in altered nitrogen metabolism and increased amino acid content in seeds. Theoretical and Applied Genetics 118, 1381–1390. 19259642 10.1007/s00122-009-0988-3

