# Supplementary material for: Integrated regulation triggered by a cryophyte ω-3 desaturase gene confers multiple-stress tolerance in tobacco
Source: J Exp Bot. 2018 Feb 8;69(8):2131–48. doi: 10.1093/jxb/ery050 (PMC6019038; doi:10.1093/jxb/ery050)
Supplement: Supplementary figures and Tables [file ery050_suppl_supplementary_figures_and_tables.pdf]

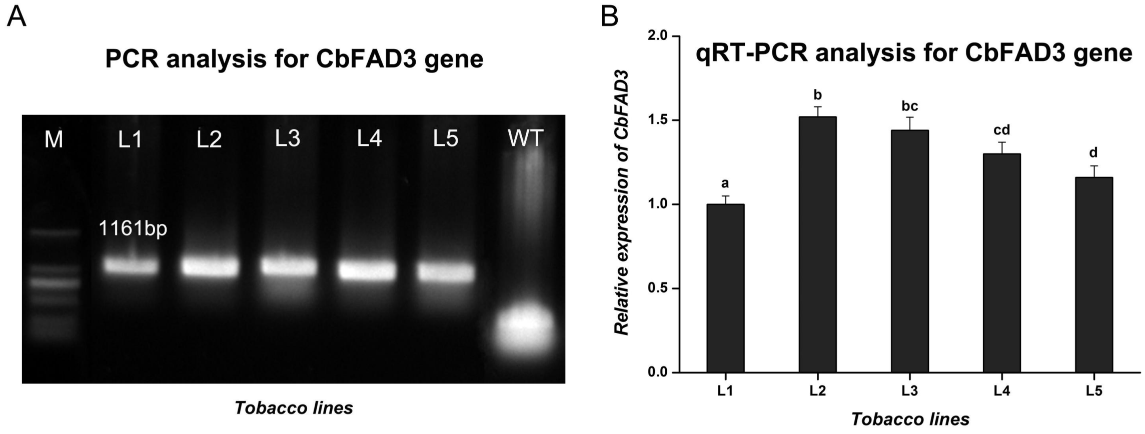

Fig. S1. Molecular analysis of *CbFAD3*-overexpressing tobacco plants. (A) PCR analysis for the ORF (1161bp) of *CbFAD3* in transgenic and WT tobacco plants. (B) qPCR analysis for the relative expression of *CbFAD3* in transgenic tobacco plants Various transgenic lines were represented as L1-L5. The cDNAs of each line were prepared from five two-week-old seedlings Values are means ± SE of three biological experiments. Statistical significance between samples was indicated by different letters according to the Student *t*-test.

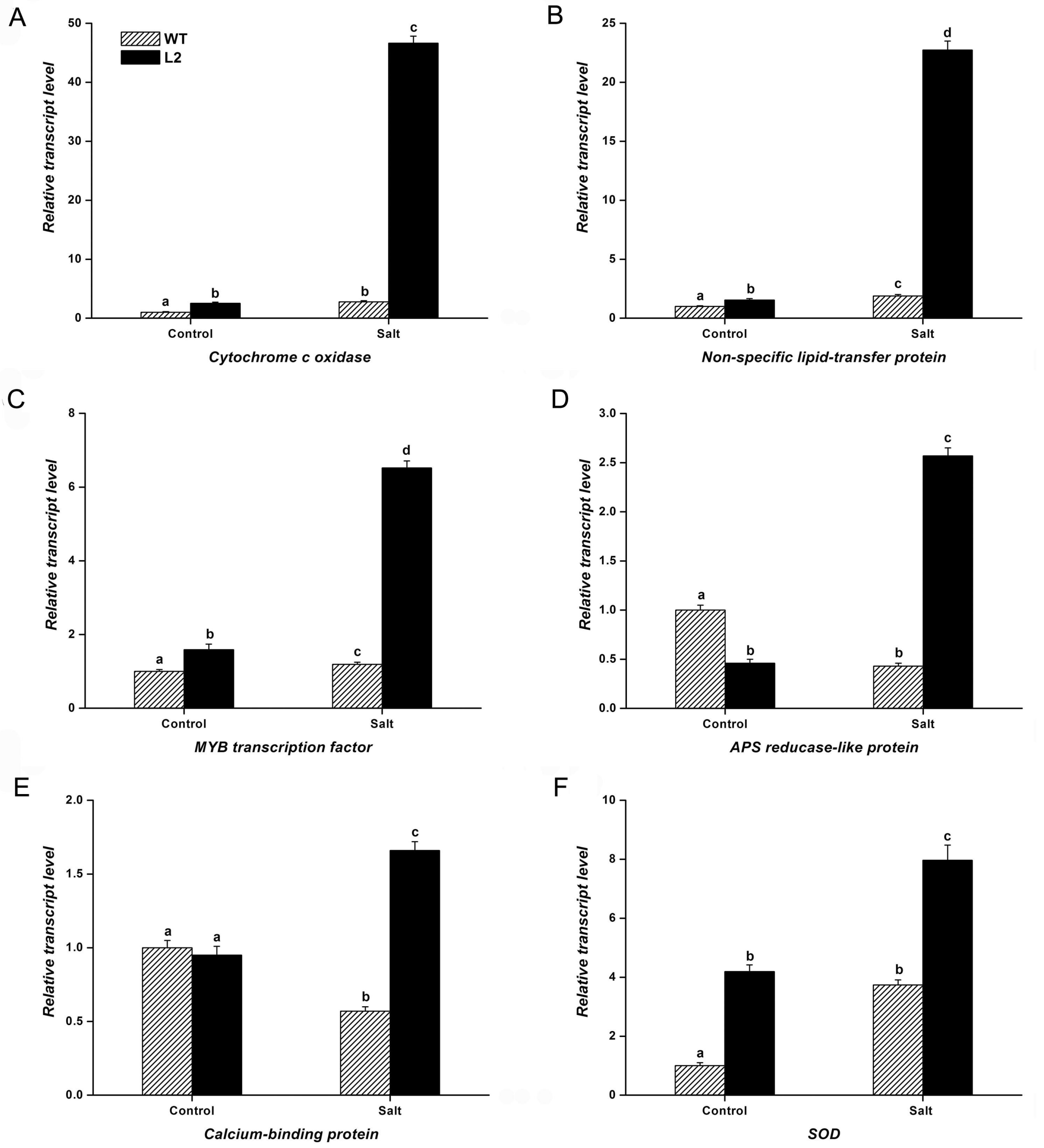

Fig. S2. qRT-PCR analysis for the increased expression of six genes in *CbFAD3*-overexpressing and WT tobacco plants under salt treatment The number of corresponding genes (A-F) in Table 2 is 1, 8, 13, 16, 23 and 39, respectively. Two-week-old seedlings of L2 and WT tobaccos were treated with 200 mM NaCl for 6 h. Leaves from five independent plants were pooled as one biology sample, and three samples were measured separately for each line. *NtL25* was used as the internal control. Values are means ± SE of three biological experiments. Statistical significance between samples was indicated by different letters according to the Student *t*-test.

Table S1. The information of primers used for gene cloning and vector construction.

Primer	annotation	Sequence (5’-3’)	Gene name	Length of production (bp)
P1	sence	GGGCGGCKATTCCTAAGCAYT	CbFAD3	424
P2	antisence	GCRAGCATGGGSAGAGGRACAG		
P3	5’outer	GAGGCACAGTGTATCTGAGCATTCGA	CbFAD3	749
P4	5’innner	GGTAAGGAACGAGGATGAAGGAGTGAAG		589
P5	3’outer	CTTCACTCCTTCATCCTCGTTCCTTACC	CbFAD3	1026
P6	3’innner	TGAAAACGACGAGTCCTGGGTTCCGCTA		927
P7	sence	GAAGGAGTAGAAAATCAAACCCTTTCT	CbFAD3	1527
P8	antisence	AAGCAGTGGTATCAACGCAGAGTAC		
P13	sence	GGATCCACCATGGTTGTTGCAATGGACCA	CbFAD3	1176
P14	antisence	GAGCTCCTAATTGATTTTAGATTTGTCAG		
P15	sence	GCTCTAGAATGGTTGTTGCAATGGACCA	CbFAD3	1176
P16	antisence	CGAGCTCCTAATTGATTTTAGATTTGTCAG		
P17	sence	ATGGTTGTTGCAATGGACCAACGCAGC	CbFAD3	1161
P18	antisence	CTAATTGATTTTAGATTTGTCAGAAGC		

Table S2. The accession number of ω-3 FADs included in alignment.

Name	Accession number	Source	Definition
AtFAD3	NP180559	*Arabidopsis thaliana*	Microsomal fatty acid desaturase
AtFAD7	P46310	*Arabidopsis thaliana*	Plastidial fatty acid desaturase
AtFAD8	P48622	*Arabidopsis thaliana*	Plastidial fatty acid desaturase
BjFAD3	ADJ58019	*Brassica juncea*	Microsomal fatty acid desaturase
BnFAD3	NP001302640	*Brassica napus*	Microsomal fatty acid desaturase
BoFAD3	AGH20189	*Brassica oleracea*	Microsomal fatty acid desaturase
CbFAD3	AKN35208	*Chorispora bungeana*	Microsomal fatty acid desaturase
DsFAD3	ABK91879	*Descurainia sophia*	Microsomal fatty acid desaturase
GmFAD3	NP001237507	*Glycine max*	Microsomal fatty acid desaturase
LeFAD3	ABX24525	*Lycopersicon esculentum*	Microsomal fatty acid desaturase
LuFAD3	AFJ53089	*Linum usitatissimum*	Microsomal fatty acid desaturase
NtFAD3	P48626	*Nicotiana tabacum*	Microsomal fatty acid desaturase
NtFAD7	D79979	*Nicotiana tabacum*	Plastidial fatty acid desaturase
OsFAD8	AAW32557	*Oryza sativa*	Plastidial fatty acid desaturase
SaFAD3	AHA05997	*Sinapis alba*	Microsomal fatty acid desaturase
TaFAD3	BAA28358	*Triticum aestivum*	Microsomal fatty acid desaturase

Table S3. The information of primers used for qRT-PCR.

Primer	annotation	Sequence (5'-3')	Gene name	Length of production (bp)	Amplification efficiency (%)
P9	sence	CCACGAAGGCAGCGAAA	CbFAD3	96	99
P10	antisence	TAGCGACCAAACTCTCAACCAA			
P11	sence	ATACGCTCTTCCACACGCTATT	CbACT	125	93
P12	antisence	TCACGATTTCACGCTCTGCT			
P19	sence	CCCCTCACCACAGAGTCTGC	NtL25	51	108
P20	antisence	AAGGGTGTTGTTGTCCTCAATCTT			
P21	sence	ACAAGGGTCCAAGTGTAGTGAAGG	Cytochrome c oxidase	60	106
P22	antisence	CCGCAACCATGCCAAGAGT			
P23	sence	TCATTTCAGTCATTGCGGTGTT	Non-specific lipid-transfer protein	96	97
P24	antisence	ATGGTGCTAAAGAAGCGTCCA			
P25	sence	AGAGTTGAGCTTGGACTTGGAGTT	MYB transcription factor	148	92
P26	antisence	TTGAACGTAGGGTGTCTTGGTG			
P27	sence	TATGGCTCTGGCTTTCACTTCA	APS reducase-like protein	190	91
P28	antisence	CTCAGCGTTCAATGGCTTCAC			
P29	sence	TTTGTCCTTGCCACTACTCACTTT	Calcium-binding protein	154	92
P30	antisence	AGCAACTTTCCCAGTTACGCTAA			
P31	sence	CTTACAGTTCATCCAACAACCCAA	SOD	65	103
P32	antisence	GAGCGAGACCAGTTATTCTTCCTC			

Table S4. Fatty acid composition of *S. cerevesiae* cells overexpressing *CbFAD3* grown at 20°C.

Plasmid	Fatty acid composition (mol%)						
	C14:0	C16:0	C16:1	C18:0	C18:1	C18:2	C18:3
pYES2	1.1±0.1	18.7±0.6	21.9±1.8	4.6±0.3	10.2±0.8	43.4±0.0	0.0±0.0
pYES2-*CbFAD3*	1.1±0.0	20.1±1.0	21.0±0.4	4.3±0.4	11.7±0.3	41.1±1.7	0.7±0.1

The data were measured after the induction of 2% galactose, 50μM C18:2, and 0.1% NP-40 for 3 d. Values are means ± SE of three biological experiments with two technical repeats for each experiment.
